# Comprehensive Histone Phosphorylation Analysis and Identification of Pf14-3-3 Protein as a Histone H3 Phosphorylation Reader in Malaria Parasites

**DOI:** 10.1371/journal.pone.0053179

**Published:** 2013-01-07

**Authors:** Eeshita G. Dastidar, Kristina Dzeyk, Jeroen Krijgsveld, Nicholas A. Malmquist, Christian Doerig, Artur Scherf, Jose-Juan Lopez-Rubio

**Affiliations:** 1 Biology of Host-Parasite Interactions Unit, Institut Pasteur, Paris, France; 2 Centre National de la Recherche Scientifique, Unité de Recherche Associé 2581, Paris, France; 3 European Molecular Biology Laboratory, Proteomics Core Facility, Heidelberg, Germany; 4 Department of Microbiology, Monash University, Clayton, Victoria, Australia; Bernhard Nocht Institute for Tropical Medicine, Germany

## Abstract

The important role of histone posttranslational modifications, particularly methylation and acetylation, in *Plasmodium falciparum* gene regulation has been established. However, the role of histone phosphorylation remains understudied. Here, we investigate histone phosphorylation utilizing liquid chromatography and tandem mass spectrometry to analyze histones extracted from asexual blood stages using two improved protocols to enhance preservation of PTMs. Enrichment for phosphopeptides lead to the detection of 14 histone phospho-modifications in *P. falciparum*. The majority of phosphorylation sites were observed at the N-terminal regions of various histones and were frequently observed adjacent to acetylated lysines. We also report the identification of one novel member of the *P. falciparum* histone phosphosite binding protein repertoire, Pf14-3-3I. Recombinant Pf14-3-3I protein bound to purified parasite histones. *In silico* structural analysis of Pf14-3-3 proteins revealed that residues responsible for binding to histone H3 S10ph and/or S28ph are conserved at the primary and the tertiary structure levels. Using a battery of H3 specific phosphopeptides, we demonstrate that Pf14-3-3I preferentially binds to H3S28ph over H3S10ph, independent of modification of neighbouring residues like H3S10phK14ac and H3S28phS32ph. Our data provide key insight into histone phosphorylation sites. The identification of a second member of the histone modification reading machinery suggests a widespread use of histone phosphorylation in the control of various nuclear processes in malaria parasites.

## Introduction

One of the deadliest infectious diseases in the world is caused by the protozoan parasites of the genus *Plasmodium*, with *P. falciparum* causing the most severe form of malaria. The complex life cycle of the parasite requires reciprocal transmission between the mosquito vector and the human host. The parasite's life style imposes constant developmental changes to survive within a selective and changing host environment. Hence, gene expression in the parasite is tightly regulated (reviewed in [1]). Recent studies have revealed the importance of histone post translational modification (PTM) in the regulation of gene expression in asexual intra-erythrocytic parasite [2]–[9]. In particular gene expression of clonally variant virulence gene families are controlled by specific histone acetylation and methylation marks [3], [6]. Results obtained from model organisms showed that distinct histone PTM create specific chromatin sites that influence many fundamental biological processes ranging from gene activation to DNA repair and cell division [10]. The enzymes involved in the histone mark ‘writing’ process and proteins that recognize specific histone marks (‘reader’ proteins) are current targets for small molecule intervention strategies in cancer and microbial disease [11]. In particular, histone acetylase and deacetylase have been validated as prime targets with a number of specific inhibitors capable of blocking cellular proliferation in various organisms including apicomplexan parasites [12], [13].

A phosphorylation modification can spatially and temporally regulate a target protein. Reversible histone phosphorylation has been associated with mitosis, chromosome condensation, DNA replication, transcription activation, apoptosis, cellular response to stress and DNA damage, depending on the context and site of this modification (reviewed in [14]). Often a scaffolding module binds a particular phosphorylated motif within the target protein to execute further downstream functions [15]. The 14-3-3 proteins are one such module known to bind histones in a phosphorylation dependent manner to regulate chromatin remodeling, transcription initiation, and hence gene expression (reviewed in [16]). Despite histone phosphorylation and its binding module being known to play an important epigenetic role, no systematic effort has been made to study this mark and its reading machinery in malaria parasites. Two very recent publications on global phospho-proteomic analyses reported a catalogue of phosphorylation sites of *P. falciparum* blood stage parasite proteins [17], [18]. However, histones were not enriched in this analysis and thus only a few histone phosphorylation sites were reported in one study. Phosphorylation modifications are known to be transient and present at low levels [19], [20], and many recent studies have demonstrated that enrichment for phosphopeptides from purified histones is often required for their detection and identification [17], [21].

*P. falciparum* histones contain abundant potential phosphorylation sites and a recent study showed that histones extracted from the parasite are substrates for the protein kinase CK2 *in vitro* [22]. Though none of the previous studies on histone modifications in *P. falciparum* specifically investigated phosphorylation [4], [8], [9], [17], [23], some do state that phosphosites were not detected by mass spectrometry or immunoblotting.

Here, we establish an in depth map of phosphorylation marks in *P. falciparum* asexual blood stage histones. We developed improved histone extraction protocols to obtain purer starting material while preserving phosphorylation, which enabled us to identify a large set of novel phospho-marks in *P. falciparum* asexual blood stage histone preparations. We also identified a reader protein that binds selectively to *P. falciparum* histone H3 phosphorylated on Ser28. Our results demonstrate a complex histone mark landscape in malaria parasites, suggesting extensive signalling in the regulation of chromatin-associated cellular processes.

## Materials and Methods

### Histone Extraction

**Figure 1:**
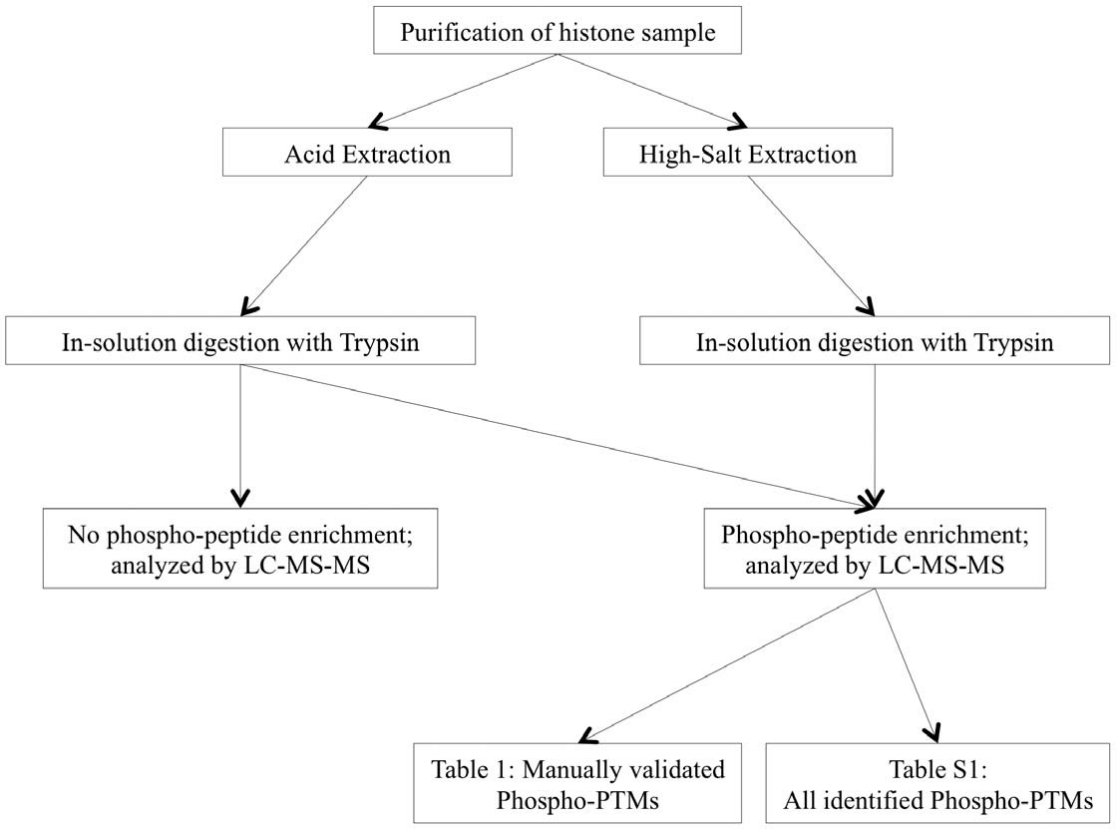
Schematic of strategies for phosphoprotein analysis of *P. falciparum* histones. Histones were purified from asynchronous culture of intra-erythrocytic malaria parasite using two improved protocols for acid extraction and high-salt extraction. Both acid and high-salt extracted histone samples were digested in-solution with trypsin. The acid extracted sample was analyzed via LC-MS/MS in two ways: either with or without phosphopeptide enrichment. A high-salt extracted sample was analyzed only after phosphopeptide enrichment. When the samples were enriched for phosphopeptides, we were able to identify 14 phosphomarks that were manually validated and are presented in Table 1. All phospho-marks detected in context with other PTMs at this step are presented in Table S1.

Histones were obtained by acid extraction and high-salt extraction techniques from unsynchronized culture of 3D7 strain parasites [24]. Parasites were grown in human blood that had been washed to deplete white blood cell contamination. Complete protease inhibitor (PI) [Roche, 11697498001] and complete phosphatase inhibitor (PPI) [Roche, 4906845001] were used during all steps, starting from collection of infected red blood cells (iRBC) through extraction of histones. All steps were performed at 4°C to minimize enzymatic activities that could potentially interfere with PTMs. For both types of extraction, 6 ml of iRBC of 5% parasitemia were collected and were lysed on ice using 0.15% saponin. The parasites were then washed three times in ice cold PBS until the supernatant was clear and no blood was observed in the parasite pellet. The resulting pellet was further treated with 0.06% saponin to remove any leftover blood contamination and washed three more times in ice cold PBS. The resulting parasite pellet was differentially treated as follows for acid and high salt extraction methods respectively (Figure 1). Red blood cells were obtained from the Etablissement Français du Sang of Necker hospital, Paris, under agreement with Institut Pasteur, and following guidelines for informed consent of donors for the use of blood or its derivatives for research purposes.

#### Acid Extraction of Histones

Histones were acid extracted using the Active Motif histone purification mini kit (cat. no. 40026) following the manufacturer's recommendations with slight modifications. Briefly, the parasite pellet was resuspended in 10 ml of ice cold Histone Extraction Buffer and sonicated for 5 minutes (30 seconds ON/OFF cycle) at 4°C using Bioruptor UCD-200 (Diagenode). The pellets were incubated overnight at 4°C to extract total histones. Cellular debris was removed by centrifugation at 16000×g at 4°C for 10 minutes. The supernatant containing crude histones was applied to a sulfopropyl (SP) resin column supplied with the kit to bind histones. The column was next washed with Histone Wash Buffer and histones were eluted using Histone Elution Buffer.

#### High-salt Extraction of Histones

The parasite pellet was resuspended in 10 ml of no-salt buffer (2 mM EDTA, 0.1 mM EGTA) and sonicated for 5 minutes (30 seconds ON/OFF cycle) at 4°C using Bioruptor UCD-200 (Diagenode). NaCl was added to a final concentration of 2 M and the sample was incubated overnight at 4°C, similar to the protocol described by [25]. Cellular debris was pelleted by centrifugation at 16000×g at 4°C for 10 minutes. The supernatant containing crude histones was then buffer-exchanged with low salt buffer (200 mM NaCl, 2 mM EDTA, 1 mM DTT) and concentrated to 1 ml volume using Ultracel-3K centrifugal filter units (Millipore, UFC800308). A packed sulfopropyl (SP) resin column supplied with the Active Motif histone purification mini kit was equilibrated with equilibration buffer (50 mM Tris-HCl, pH 8.0, 200 mM NaCl, 2 mM EDTA) and the concentrated crude histone extract was passed through this column. The column was washed three times with wash buffer (50 mM Tris-HCl, pH 8.0, 500 mM NaCl, 2 mM EDTA), and total histones were eluted in elution buffer (50 mM Tris-HCl, pH 8.0, 2 M NaCl, 2 mM EDTA).

The purified histones were run on 12% SDS-PAGE gel with MES buffer and stained with Bio-safe coomassie blue (Bio-Rad, 161-0786, detection limit 50–100 ng).

### Generation and purification of recombinant proteins

The coding region of proteins 14-3-3I (PF3D7_0818200) and 14-3-3II (PF3D7_1362100) were GST-tagged at N-terminal by cloning into pGEX vector. Primer pair used for 14-3-3I were: f69GSTn: CGCGGGATCCATGGCAACATCTGA-AGAATTAAAAC and r69GSTn: GCGCGAATTCTCATTCTAATCCTTCGTCTTTTGAT and that for 14-3-3II were: f309GSTn: GCGCGGATCCATGAATCAATATATTGATAACGATATTTC and r309GSTn: GCGCGAATTCTTATGTATGAGTACTATTCATAATGTC. The resulting constructs were named GST-14-3-3I and GST-14-3-3II respectively and were transformed into the *E. coli* strain BL21. Bacteria expressing the GST-tagged version of the 204-bp chromo-domain of heterochromatin protein 1 (GST-HP1CD) was kindly supplied by Dr. Rosaura Hernandez-Rivas [26]. Expression of GST-fusion proteins was induced with 0.50 mM IPTG at 37°C for GST-14-3-3I and GST-14-3-3II and at 30°C for GST-HP1CD for 6 hours. All the GST-fusion proteins were purified using glutathione sepharose beads (GE Healthcare Life Sciences). The purity of the eluted proteins was checked by standard SDS-PAGE and coomassie staining.

### Production of anti-14-3-3 antibody

Recombinant GST-14-3-3I protein was purified as above to produce rat anti-14-3-3I antibodies according to the standard protocols of Genscript (USA).

### Western Blot (WB) Analysis

Extracted total histones were separated by SDS-PAGE using 12% bis-tris precast gel (Bio-Rad, 345-0118) and MES buffer (Invitrogen, NP0002). The proteins were then transferred overnight at 4°C on to nitrocellulose membrane of pore size 0.20 µm in presence of 20% methanol in Nupage transfer buffer (Invitrogen, NP0006). The membrane was then blocked with 3% BSA in TBS buffer containing 0.05% Tween20 and 100 mM NaF. Commercial antibodies purchased from Abcam: anti-H3core (ab1791) [1∶2500 dilution], anti-H3S10ph (ab5176) [1∶1000 dilution], anti-H3S28ph (ab5169) [1∶1000 dilution], and anti-H3T11ph (ab5168) [1∶1000 dilution] were then used to probe the membrane. After probing with appropriate secondary antibodies conjugated to horseradish peroxidase (GE Healthcare Life Sciences), the membranes were developed with Super Signal West FEMTO Chemiluminescent Substrate (Thermo Scientific) following the manufacturer's recommendations.

Western blot analysis was performed on cytoplasmic and nuclear fractions of unsynchronized 3D7 parasites prepared as described in [3], [27]. Briefly, cytoplasmic and nuclear extracts were separated and transferred onto nitrocellulose membrane by standard SDS-PAGE protocol. The membranes were then blocked overnight at 4°C using blocking buffer containing 5% milk in TBS containing 0.25% Tween-20. The membranes were subsequently incubated with anti-14-3-3I antibody diluted in blocking buffer [1∶1000 dilution]. Detection with the secondary antibody was as described above.

### Immunofluorescence Assay (IFA)

IFA was performed on synchronized stages (Ring, Trophozoite, and Schizont) of the wild type 3D7 strains as described in [28]. The fixed cells were incubated with primary antibody anti-14-3-3I [1∶100 dilution]. After incubating with appropriate secondary antibodies, the cells were examined with a Nikon microscope.

### Mass spectrometry

Histones purified from unsynchronised 3D7 parasites were digested and analysed by liquid chromatography-tandem mass spectrometric (LC-MS/MS) for the detection and site-localization of phosphorylation.

#### Sample preparation and tryptic digestion

Prior to in-solution digestion, the samples containing 5–10 µg protein were concentrated with Amicon 3 kDa MWCO filters (Millipore) and the buffer was simultaneously exchanged for 50 mM Ammonium bicarbonate for subsequent trypsin digestion. Proteins were reduced with DTT and alkylated with iodacedamide. Incubation with trypsin (1 µg enzyme/50 µg protein) was carried out overnight at 37°C. The reaction was stopped by adding 5 ul of 1% formic acid. Phos-TiO phosphopeptide enrichment kit (GL Sciences) was used in order to enrich for phosphopeptides according to the manufacturer's recommendations. After elution with 5% Ammonium hydroxide, the phosphopeptides were dried down in a speed vacuum centrifuge. Prior to mass spectrometry analysis, the peptides were reconstituted in 5 µL 0.5% formic acid.

#### LC-MS/MS

Peptides were separated using the nanoAcquity UPLC system (Waters) fitted with a trapping (nanoAcquity Symmetry C18, 5 µm, 180 µm×20 mm) and an analytical column (nanoAcquity BEH C18, 1.7 µm, 75 µm×200 mm). The outlet of the analytical column was coupled directly to an LTQ Orbitrap Velos (Thermo Fisher Scientific) using the Proxeon nanospray source. Solvent A was water, 0.1% formic acid and solvent B was acetonitrile, 0.1% formic acid. The samples (4 µL) were loaded with a constant flow of solvent A at 15 µL/min onto the trapping column. Peptides were eluted via the analytical column at a constant flow of 0.3 µL/min in a 30-min linear gradient from 3% to 40% solvent B. The peptides were introduced into the mass spectrometer via a Pico-Tip Emitter 360 µm OD×20 µm ID; 10 µm tip (New Objective) and a spray voltage of 2.1 kV was applied. The capillary temperature was set at 230°C. Full scan MS spectra with mass range 300–1700 m/z were acquired in profile mode in the FT with resolution of 30000. The filling time was set at maximum of 500 ms with limitation of 106 ions. The most intense ions (up to 15) from the full scan MS were selected for sequencing in the LTQ. Normalized collision energy of 40% was used, and the fragmentation was performed after accumulation of 3×10^4^ ions or after filling time of 50 ms for each precursor ion (whichever occurred first). MS/MS data were acquired in centroid mode with resolution of 7500. Charge state screening was enabled and only doubly and triply charged precursor ions were selected for MS/MS. The dynamic exclusion list was restricted to 500 entries with maximum retention period of 30 s and relative mass window of 7 ppm. For internal mass calibration, a lock mass correction using a background ion (m/z 445.12003) was applied.

#### Data analysis

Software Max Quant (version 1.2.0.18) was used for filtering the data and creating .mgf files needed for searching in MASCOT version 2.2.03 (Matrix Science). The data were searched against a user-compiled database comprising 3200 human proteins, including histones, to which *Plasmodium* histones were added. The data were searched for acetylation (K), mono- and di-methylation (R), mono-, di- and tri-methylation (K), oxidation (M) and phosphorylation (STY) as variable modifications and carbamidomethylation (C) as fixed modification. The mass error tolerance for the full scan MS spectra was set at 10 ppm and for the MS/MS spectra at 0.5 Da. A maximum of 3 missed cleavages was allowed. The .dat files were loaded into Scaffold (version 3.00.06) and the phosphopeptides with Mascot score above 20 were reported. Site localisation was determined by MaxQuant, requiring a site probability score >0.75 and a difference score >5. See Figures S3, S4, S5, S6, S7, S8, S9, S10, S11, S12, S13, S14, S15, S16, S17, S18, S19, S20, S21 for annotated mass spectra of the identified modifications.

### ELISA-based protein binding assay

Binding of GST-14-3-3I and GST-14-3-3II proteins to purified parasite histones and phospho-modified histone peptides was checked in ELISA assay. Maxisorp NUNC ELISA plates were used for all the assays. Buffer A (50 mM HEPES, pH 8.0, 150 mM NaCl, 1 mM CaCl2, 1 mM MgCl2, 1 mM DTT, 100 mM NaF, 0.005% Tween-20, 1% BSA, and 10% Glycerol) was used as binding and washing buffer unless mentioned otherwise.

#### Purified parasite histones and 14-3-3 protein binding assay

Core histones were purified from unsynchronized 3D7 parasites (see above) and diluted in Tris pH 8.0 at 0.40 µg/100 µl and coated onto an ELISA plate in duplicates overnight at 4°C. The following day the plate was washed three times 3 minutes each in a buffer containing 50 mM HEPES and 150 mM NaCl. GST-14-3-3I and GST-14-3-3II [1.0 µg/100 µl per well], GST-HP1CD or GST [0.50 µg/100 µl per well] diluted in buffer A were added to the wells and incubated at room temperature for 2 hours. The protein solutions were removed and the wells were then washed three times 3 minutes each in buffer A. The plate was next incubated at room temperature for 1 hour with anti-GST-HRP antibody diluted in buffer A [1∶1000], and the wells were then washed three times 3 minutes each in buffer A. The wells were finally fluorogenically developed with Amplex Ultra Red reagent (Invitrogen; A36006) as per the manufacturer's recommendations and read using a Safas spectrophotometer.

#### Biotinylated histone peptides and 14-3-3 protein binding assay

Biotinylated peptides were ordered from Biomatik (USA) (see Table 1 for peptide details). Maxisorp ELISA plates were coated with streptavidin [1.0 µg/100 µl per well] overnight at 4°C. The plate was then blocked in buffer A for 1 hour at room temperature. After removing the blocking buffer, appropriate wells in the plate were incubated with pertinent peptides [0.50 µg/100 µl dilution per well] in triplicates for 1 hour at room temperature to ensure complete binding. Subsequently, the peptide solutions were removed from the wells and the entire plate was washed three times 3 minutes each in buffer A to remove any unbound peptides. The plate was then incubated with GST-14-3-3I and GST-14-3-3II [1.0 µg/100 µl per well], GST-HP1CD and GST [0.50 µg/100 µl per well] diluted in buffer A at appropriate wells containing different peptides. All the proteins of same dilutions as above were also put into wells that were streptavidin coated but not incubated with peptides, to determine if any of the proteins bound directly to streptavidin. After incubating at room temperature for 2 hours, the protein solutions were removed, and the plate was washed three times 3 minutes each in buffer A. The plate was next incubated at room temperature for 1 hour with anti-GST-HRP antibody diluted in buffer A [1∶5000], following which the wells were washed three times 3 minutes each in buffer A. One streptavidin coated well which was not incubated with any peptides or proteins, was incubated with anti-GST-HRP antibody to determine any direct interaction between streptavidin and anti-GST-HRP antibody. All the wells were then fluorogenically developed with Amplex Ultra Red reagent (Invitrogen; A36006) as per manufacturer's recommendation and quantified using a Safas spectrophotometer. All experiments were performed in triplicate.

**Table 1:** List of histone phospho-modifications identified in this study using MaxQuant requiring site a probability score >0.75 and a difference score >5.

Histone	Peptide	Modification	MRcalc	m/z	Mascot Score	Annotation
H2A	K.GTSNSAKAGLQFPVGR.I	Phospho: 5	1668.80	835.41	63	S18ph
	K.KSQLKAGTANQDY.-	Phospho: 2	1502.67	752.35	39	S120ph*
	K.KSQLKAGTANQDY.-	Phospho: 8	1374.58	688.30	31	T126ph*
H2A.Z	K.VLGLGKGGKGKTGSGKTK.K	Phospho: 14	1920.01	961.01	58	S32ph
H2B.Z Also known as H2Bv†	M.SGKGPAQKSQAAKK.T	Acetyl: 3, 8, 13, Phospho: 1	1590.78	796.40	38	S1ph, K3ac, K8ac, K13ac
H3.1	R.KSTAGKAPRK.Q	Acetyl:1, 6, Phospho: 2	1078.52	540.27	31	K9acS10ph K14ac
	R.KSTAGKAPR.K	Acetyl:1, 6, Phospho: 3	1078.52	540.27	34	K9acT11ph K14ac
	R.KSAPISAGIK.K	Phospho: 2	1050.55	526.28	63	S28ph
	R.KSAPISAGIK.K	Phospho: 2, 6	1130.51	566.26	69	S28phS32ph*
	R.YQKSTDLLIR.K	Phospho: 4	1315.65	658.83	51	S57ph
H3.3	R.KSAPVSTGIK.K	Phospho: 2	1066.54	534.28	65	S28ph*
	R.KSAPVSTGIK.K	Phospho: 6	1066.54	534.28	47	S32ph*
	R.KFQKSTDLLIR.K	Phospho: 5	1427.75	476.93	59	S57ph*
H3.1/H3.3	R.KQLASKAAR.K	Acetyl: 6; Phospho: 5	1093.56	547.79	42	S22phK23ac

* Histone phospho-modifications identified in Treeck et al., 2011. For other histone phospho-modifications identified in that study see bottom Table S1.

† Talbert PB, Ahmad K, Almouzni G, Ausio J, Berger F, et al. (2012) A unified phylogeny-based nomenclature for histone variants. Epigenetics Chromatin 5∶7.

### *In silico* modelling of Pf14-3-3 proteins

Amino acid sequences of Pf14-3-3I and Pf14-3-3II were submitted to the I-TASSER server for structural prediction [29], [30]. Protein structures were visualized using MacPyMol version 0.99rc6. Sequence alignments of Pf14-3-3I and Pf14-3-3II to 14-3-3 proteins from human (NP_003397), *Nicotiana tobaccum* (P93343), and *Cryptosporidium parvum* (cdg3_1290) were performed using ClustalW2 and visualized using BOXSHADE.

## Results

### Development of improved acid and high-salt purification methods for *P. falciparum* histone phosphoprotein analysis

**Figure 2:**
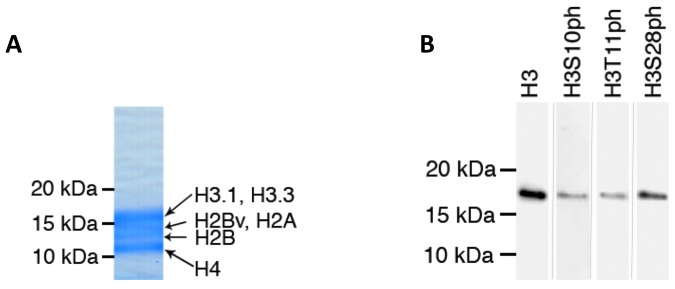
Improved extraction methods preserve histone phosphorylation. A) Coomassie-stained gel demonstrating the purity of extracted histone sample by acid extraction protocol. The high-salt extraction protocol yields similar high purity sample (data not shown). B) Western blot analysis performed on acid extracted histone with commercially available antibodies against H3 core, H3S10ph, H3T11ph, and H3S28ph modifications. These antibodies yielded a single band corresponding to the expected size of histone H3 (∼17 kDa) when developed with Super Signal West FEMTO Chemiluminescent Substrate.

Previous studies on histone modifications in *P. falciparum* [8], [23] did not detect any phosphorylation marks on histones (reviewed in [31]). Most of these studies relied on traditional acid extraction method to partially purify parasite histones and did not include affinity enrichment of the phosphopeptides [17], [21], [32], [33]. We developed two methods to yield purer histones while maintaining phospho-marks and included a phosphopeptide enrichment step in our study (Figure 1). A typical purified histone sample obtained by acid extraction is shown in Figure 2A. To confirm that the samples had retained their phospho-PTMs, we probed our purified histone samples with commercially available antibodies against the H3S10ph, H3T11ph, and H3S28ph modifications of histone H3, which is highly conserved between *P. falciparum* and human. Western blot analysis performed with these antibodies on histones extracted by either acid (Figure 2B) or high-salt extraction method (data not shown) yielded a single band corresponding to the expected size of histone H3 (∼17 kDa), demonstrating that the analyzed phosphorylation sites are preserved by our histone extraction methods.

### LC-MS/MS analysis of histone samples enriched for phosphopeptides revealed the presence of 14 histone phosphorylation sites

Purified histones obtained by both methods were analysed by LC-MS/MS after in-solution trypsin digestion (Figure 1). A portion of the acid-extracted sample was directly analysed by LC-MS/MS. This analysis identified three phospho-modified residues on both H3.1 and H3.3, namely Ser-28, Ser-32, and Thr-45 (data not shown), indicating that these marks may represent the most abundant phospho-modifications in the samples analyzed. Importantly, we also identified many other histone modifications (e.g. methylation) present in their physiological combinations on the purified histones (data not shown).

Enrichment for phosphopeptides on the trypsin-digested histone samples enabled us to identify 14 phospho-modifications in both the acid and high-salt extracted histone samples (Table 1). The analyses for phospho-enriched samples were performed four times on two biologically distinct acid-extracted samples and once on the salt-extracted sample (Figure 1). Although Table S1 displays all the phosphopeptides identified in all five analyses, only the modifications identified on *P. falciparum* specific peptides (peptide sequences unique for *P. falciparum*) are taken into account for further consideration to prevent including any data from possible human contaminants. We identified phosphorylation sites distributed on all histones with the exception of H4; for one modification, we could not specify the histone variant given the sequence conservation between them for the identified peptide (Table 1 and Figure S1). Multiple modifications on the same peptide were also observed in the phospho-enriched samples (Table 1 and S1).

### Pf14-3-3I selectively binds to H3S28ph

**Figure 3:**
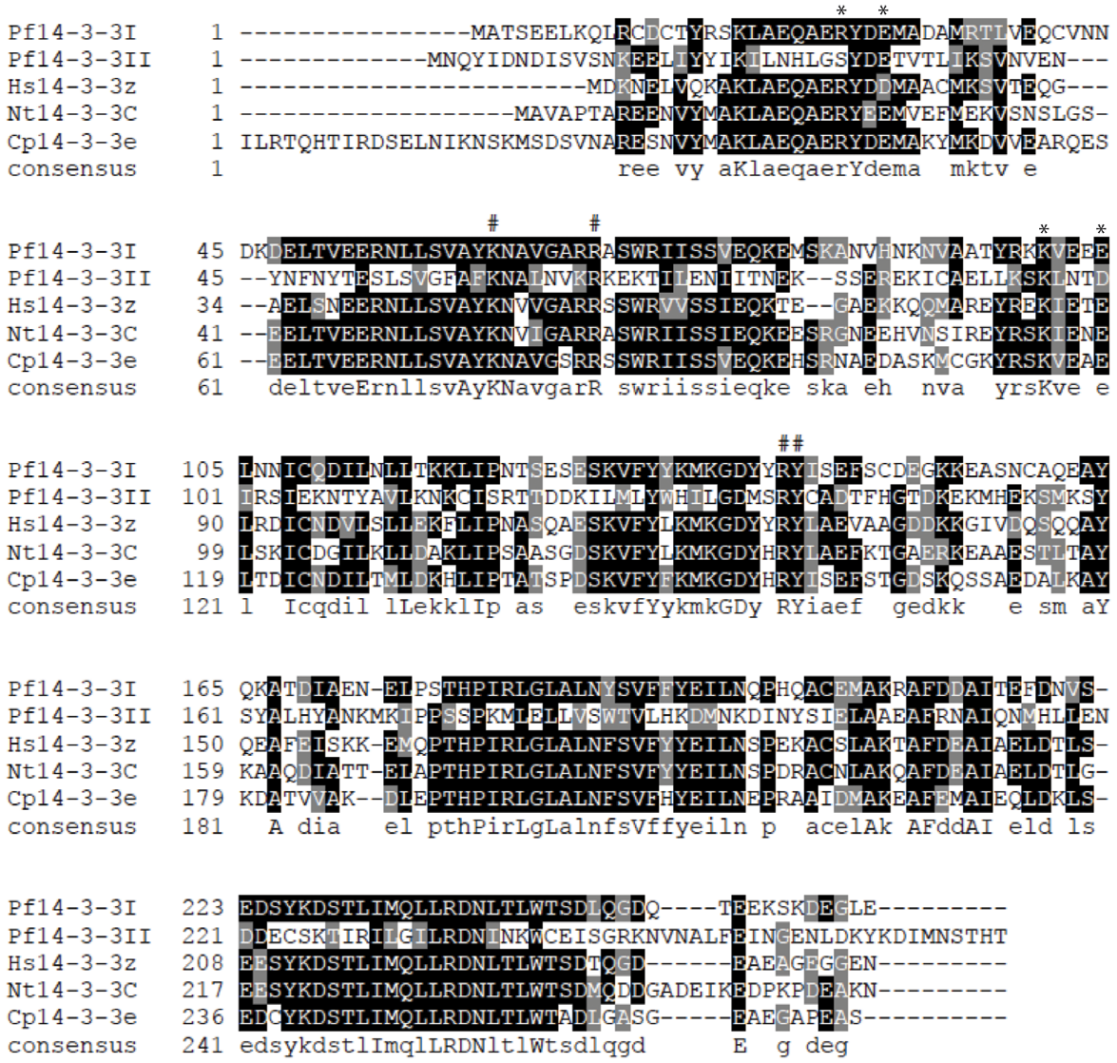
Sequence alignment of 14-3-3 proteins. Amino acid sequences of Pf14-3-3I (PF3D7_0818200), Pf14-3-3II (PF3D7_1362100), human 14-3-3 zeta (NP_003397), *Nicotiana tobaccum* 14-3-3-like protein C (P93343), and *Cryptosporidium parvum* epsilon (cdg3_1290) aligned by ClustalW2. Residues involved in the binding of phosphorylated residues are marked with (#). Residues involved in stabilizing homo- or hetero-dimerization are marked with (*).

Following the discovery of an array of histone phospho-modifications in intra-erythrocytic parasites, we next investigated how the histone phosphorylation marks are ‘read’ by the nuclear machinery. Previous studies have shown that histone modifications can recruit various proteins to perform effector functions. Proteins containing 14-3-3 domains bind phosphoserines of histones (reviewed in [34], [35]). Three putative 14-3-3 proteins are predicted in *P. falciparum* (PF3D7_0818200, PF3D7_1362100, and PF3D7_1422900), of which the first two are expressed at higher levels in the asexual stage parasite; we therefore focussed our attention on these proteins. Pf14-3-3I and Pf14-3-3II amino acid sequences were aligned with that of human (NP_003397), *Nicotiana tobaccum* (P93343), and *Cryptosporidium parvum* (cdg3_1290) 14-3-3 proteins revealing approximately 70–80% and 25% similarity of Pf14-3-3I and Pf14-3-3II to these model 14-3-3 proteins, respectively (Figure 3). Residues involved in phosphoserine recognition [36], [37] are conserved in both plasmodial proteins (Figure 3).

**Figure 4:**
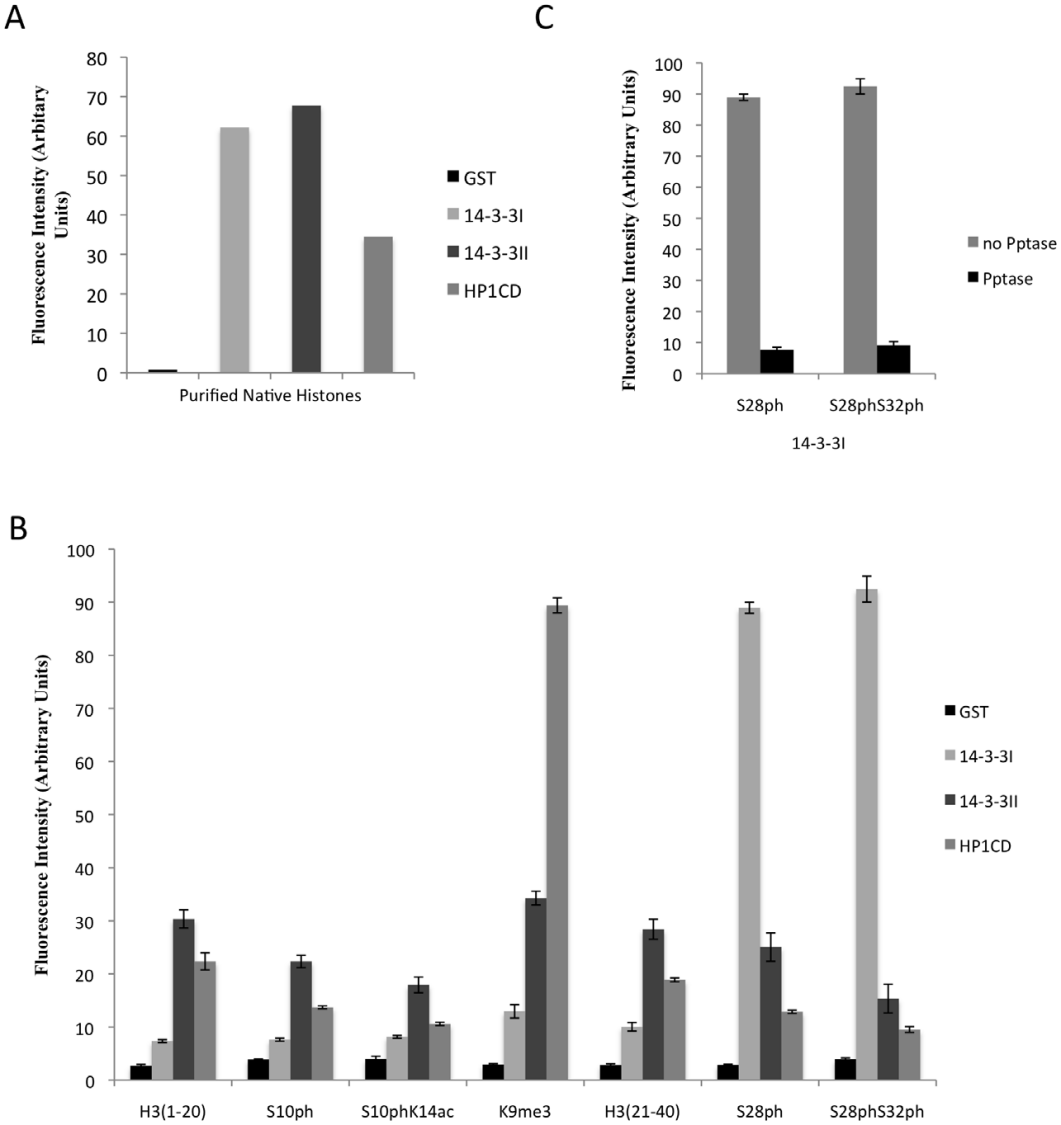
14-3-3 protein binding studies to native histones and phosphorylated histone H3 peptides. A) Interaction between purified histone sample and GST-tagged recombinant Pf14-3-3I, Pf14-3-3II, and Pf-HP1-CD was observed by ELISA-based binding assay. B) Binding of GST-14-3-3I and GST-14-3-3II to different synthetic peptides listed in Table 2 was tested by ELISA-based binding assay. C) ELISA-based binding assay was performed with GST-14-3-3I and phosphatase treated and untreated H3S28ph and H3S28phS32ph peptides.

We next expressed recombinant GST-tagged versions of Pf14-3-3I and Pf14-3-3II to experimentally validate the predicted function of these proteins. Purified GST fusion proteins were used in an ELISA-based binding assay to determine their ability to bind purified parasite histones. The only member of the *P. falciparum* histone code reading machinery described to date, PfHP1, binds to H3K9me3 via its chromo domain [26], [38], providing a positive control for these experiments. Purified GST protein was used as a negative control. As expected, GST-HP1CD bound to the purified parasite histones, while GST alone did not. Both the putative 14-3-3 proteins, GST-14-3-3I and GST-14-3-3II clearly bound purified parasite histones (Figure 4A). This result indicates that both the Pf14-3-3 proteins, like the PfHP1 chromo domain, are indeed able to interact with purified parasite histones.

Next, we determined which specific phosphorylation site(s) are responsible for 14-3-3 recognition. Proteins containing 14-3-3 domains are known to bind histone H3 phosphorylated at Ser-10 and/or Ser-28 residues [36], [39], [40]. Binding of GST-14-3-3I and GST-14-3-3II to different synthetic peptides, either unmodified, trimethylated at H3K9, or phosphorylated at positions H3S10 and H3S28 (Table 2), was measured by ELISA. Since adjacent histone modifications are known to affect binding of a protein to a particular modification [41], we included two dually modified peptides, H3S10phK14ac and H3S28phS32ph (Table 2), which we had observed in our mass spectrometry analysis on purified parasite histones (Table 1). GST-HP1CD was used as positive control. Clear binding of GST-HP1CD to the H3K9me3 peptide was observed, while it did not bind unmodified H3^1–20^ peptide or any of the other synthetic peptides used in this study (Figure 4B). Likewise, GST-14-3-3I clearly bound H3S28ph and H3S28phS32ph peptides (figure 4B). Much lower levels of binding were observed between GST-14-3-3I and unmodified H3^1–20^, unmodified H3^21–40^, H3K9me3, H3S10ph or dually modified H3S10phK14ac peptides. Though GST-14-3-3II protein clearly bound purified parasite histones, it did not bind any of the peptides used in this binding assay to a level comparable to that with which GST-HP1CD bound H3K9me3 or GST-14-3-3I bound H3S28ph and H3S28phS32ph peptides. We detected low level binding of Pf14-3-3II to all the peptides used in this study.

**Table 2:** List of biotinylated H3-peptides used for ELISA binding assay.

synthetic peptide name	histone H3 amino acid sequence
H3^1–20^	ARTKQTARKSTAGKAPRKQL-K(biotin)
H3S10ph	ARTKQTARKS(ph)TAGKAPRKQL-K(biotin)
H3S10phK14ac	ARTKQTARKS(ph)TAGK(ac)APRKQL-K(biotin)
H3K9me3^1–20^	ARTKQTARK(tri-methylated)STAGKAPRKQL-K(biotin)
H3^21–40^	ASKAARKSAPISAGIKKPHR-K(biotin)
H3S28ph	ASKAARKS(ph)APISAGIKKPHR-K(biotin)
H3S28phS32ph	ASKAARKS(ph)APIS(ph)AGIKKPHR-K(biotin)

We used a similar ELISA approach to confirm that the observed binding of GST-14-3-3I to phosphorylated peptides H3S28ph and H3S28phS32ph was indeed due to phosphorylation. 0.5 µg phosphorylated H3S28ph and H3S28phS32ph peptides were bound to the plate. H3K9me3 peptide was used as control peptide. All the peptides were then treated with λ-phosphatase (Pptase) [NEB, P0753S]. Control wells with same peptides were incubated with phosphatase reaction buffer without λ-phosphatase. Binding of GST-14-3-3I to both the H3S28ph and H3S28phS32ph peptides was greatly reduced when the peptides were phosphatase-treated, while clear binding was observed when no phosphatase was added to the peptides (Figure 4C). In a similar ELISA based assay, the same peptides were probed with anti-H3S28 antibody, after being incubated with or without phosphatase. The phosphorylation signal was greatly reduced after phosphatase treatment (data not shown). The control peptide H3K9me3 was similarly incubated with or without phosphatase and binding of GST-HP1CD to the peptide after the treatment was determined. No significant reduction in binding of GST-HP1CD to the H3K9me3 peptide being observed after phosphatase treatment (data not shown).

### 14-3-3I is present in both cytoplasmic and nuclear compartments of the intra-erythrocytic parasite

**Figure 5:**
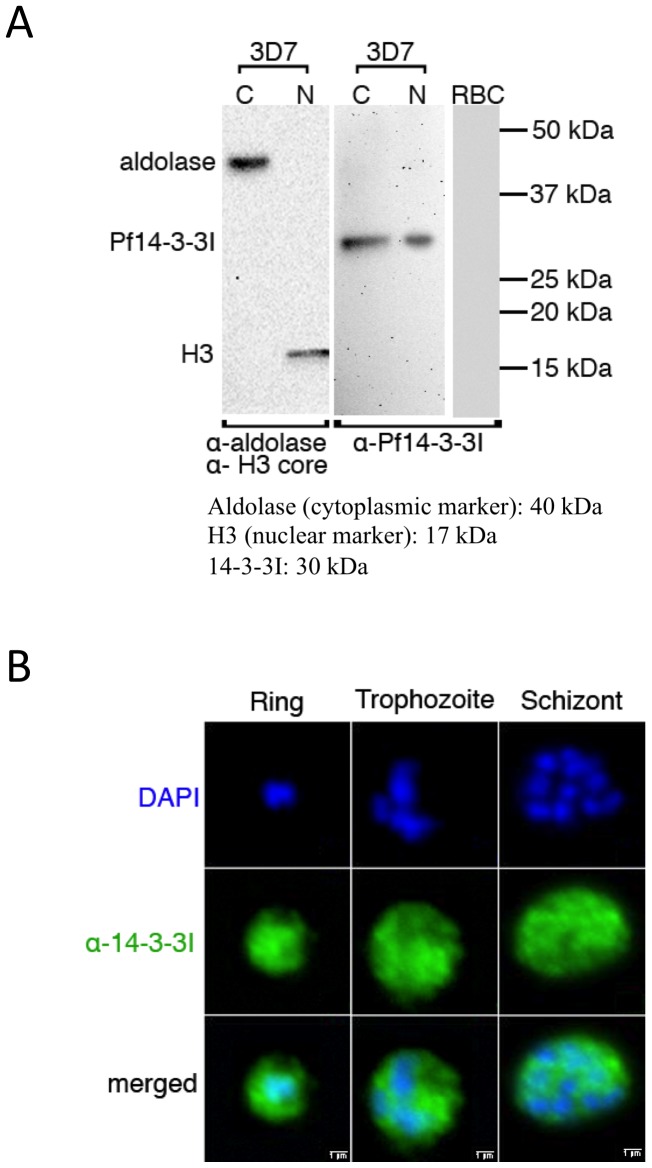
Subcellular location of 14-3-3 in asexual blood stage parasites. A) Cellular localization of Pf14-3-3I was investigated by probing cytoplasmic and nuclear fraction prepared from asynchronous 3D7 parasite culture with anti-14-3-3I antibody in western blot analysis. Aldolase and histone H3 antibodies were used to check the purity of cytoplasmic and nuclear fraction respectively. Protein extract from non infected red blood cells (RBC) was used as control to show that anti Pf14-3- antibody does not recognized mammalian homologues present in human erythrocytes. B) Using anti-14-3-3I antibody in immunofluorescence assay, the Pf14-3-3I protein was localized in both nuclear and cytoplasmic compartments.

Previous studies identified 14-3-3 protein in *P. berghei*, *P. knowlesii* and *P. falciparum* in total extracts [42]–[44], but no data on subcellular localization are available. An anti-14-3-3I antibody was generated against the full-length GST fusion protein and was used in western blot to probe cytoplasmic and nuclear extracts from unsynchronised 3D7 parasites. A single band corresponding to expected size of the protein (∼30 kDa) was observed in both fractions, whereas it did not recognize mammalian isoforms present in human erythrocytes, indicating the antibody was specific for Pf14-3-3I and the protein was present in both cytoplasmic and nuclear compartments of the parasite (Figure 5A). Subsequently, the same antibody was used in IFA to visualize the location of the protein throughout the life cycle of intra-erythrocytic parasite (Figure 5B). An IFA signal was observed in cytoplasmic compartment and overlapping with the nuclear signal at all asexual stages of the parasite, which is compatible with the protein immunoblot results. Since 14-3-3 proteins have been reported to be able to bind a multitude of functionally variant proteins and not only histones in other eukaryotic organisms [35], [45], [46], the presence of the protein in different cellular compartments is consistent with its probable pleiotropic role of these proteins in *P. falciparum*.

### *In silico* structural analysis of Pf14-3-3 proteins revealed conservation of residues responsible for phosphoserine binding

**Figure 6:**
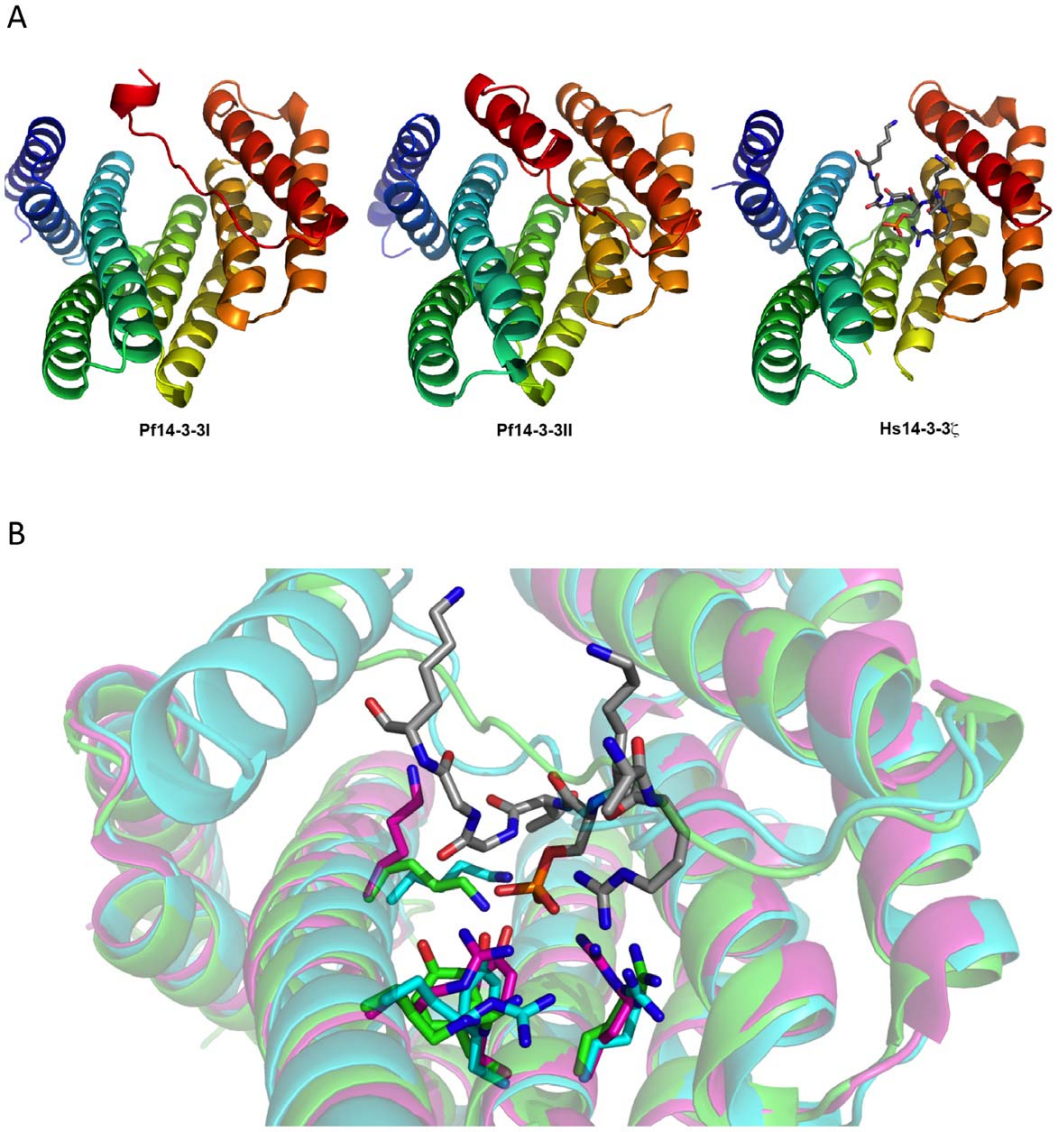
Homology-based structural models of Pf14-3-3 proteins. A) The highest scoring models of Pf14-3-3I and Pf14-3-3II are displayed alongside the structure of human 14-3-3 zeta co-crystallized with phosphorylated histone (H3S10ph) peptide. Ribbon diagrams are coloured blue to red from their N- to C-termini. The phosphorylated histone peptide in the human structure is coloured gray for carbon, blue for nitrogen, red for oxygen, orange for phosphate. B) The above Pf14-3-3I structure (green), Pf14-3-3II structure (cyan), and the human 14-3-3 zeta structure co-crystallized with an ARKSphTGGK peptide (magenta, from 2C1N). The four residues involved in phosphorylated residue binding for each 14-3-3 protein are displayed as sticks. The phosphoserine side-chain from the bound peptide in the human structure is also displayed as sticks. Nitrogen is blue, oxygen is red, phosphate is orange, and carbon is gray.

Pf14-3-3I and Pf14-3-3II amino acid sequences were submitted to the I-TASSER server for protein structure prediction [29], [30]. The returned sequence alignments and structural analogues were exclusively 14-3-3 proteins from other organisms, including human, tobacco, and *Cryptosporidium parvum*. The I-TASSER server also predicted five structural models for each of the two *P. falciparum* 14-3-3 proteins. The highest scoring models of Pf14-3-3I and Pf14-3-3II are displayed alongside the structure of human 14-3-3 zeta co-crystallized with phosphorylated histone (H3S10ph) peptide in Figure 6A. As predicted from the primary protein sequence alignments, all the Pf14-3-3 structural models revealed the lysine, arginine, and tyrosine amino acid side chains involved in phosphoserine recognition to be positioned similarly to those in the solved 14-3-3 protein structures from model organisms [36], [37] (Figure 6B). The backbone chains of all ten structural models were highly structurally similar with the exception of the location of the C-terminal tail. Of the five models predicted for Pf14-3-3I (Figure 6A), one included C-terminal residues occupying the putative phosphoprotein binding site, while in the other four models the phosphoprotein binding site was unoccupied (Figure S2A). The Pf14-3-3I C-terminal segment occupying the phosphoprotein binding site makes no apparent polar contacts with any of the residues implicated in phosphoserine binding. Conversely, all five Pf14-3-3II predicted structural models included C-terminal residues in the phosphoprotein binding site (Figure 6A and S2B). In one of these models, Asn-251 from the C-terminal segment makes a polar contact with the Tyr-139 residue implicated in phosphoserine recognition. This variable occupancy of the phosphoprotein binding site of Pf14-3-3I, together with the indication of a polar interaction in this site in Pf14-3-3II, suggest this site may indeed be partially occupied by the C-terminus of the purified parasite proteins.

## Discussion

Nucleosome modifications, together with specific proteins recruited to these modifications (histone readers), dictate many fundamental chromatin-associated processes in eukaryotes. This field is now emerging as a fascinating research area in *Plasmodium*, and is clearly linked to virulence gene control in this organism. Here, we have performed an in depth analysis of histone phosphorylation of asexual blood stage parasites of *P. falciparum*. To this end, we have developed improved methods of extracting histone samples that retain unprecedented levels of PTMs. Our analysis of phospho-enriched histone peptides revealed multiple phosphorylation sites mostly at the N-terminal region of most histones. These marks are frequently seen in combination with neighbouring lysine acetylation (and methylation). In addition, we identified Pf14-3-3I as a phospho histone mark binding protein. Previously, we and others had identified heterochromatin protein 1 (PfHP1) binding to H3K9 methylated as a key mediator in heterochromatin formation linked to the expression of clonally variant gene families [26], [38]. Pf14-3-3I is the second *P. falciparum* histone mark reader protein to be identified.

Phosphorylation of histones plays a role in cell signalling and transcriptional regulation in a number of eukaryotic organisms (reviewed in [14]). Plasmodial histones contain abundant serine, threonine and tyrosine residues for potential phosphorylation. Although previous studies have identified the role of histone methylation and acetylation in plasmodial gene regulation, histone phosphorylation was not reported in these studies [2]–[9]. In these studies, traditional methods of acid extraction were used to obtain partially purified proteins for further phospho-protein analysis. [8], [23]. However, the labile nature of phospho-marks and the relatively low abundance of most phospho-modifications may explain the negative results in previous reports on histone marks [19], [20]. For this reason, we combined improved purification methods of histones with phosphopeptide enrichment to revisit this topic [17], [21], [32], [33]. We improved on two traditional histone extraction protocols, namely acid extraction and non-acid high-salt extraction [25], to better preserve PTMs including phosphorylation. Using commercially available antibodies we were able to demonstrate the retention of various phospho-modifications in the histone samples prepared by either method. All samples were initially analyzed by LC-MS/MS, without enriching for phosphopeptides. This step enabled us to identify many PTMs with a significant mascot score, which were not manually validated (data not shown). We were also able to identify multiple modifications on the same peptide, which supports a possible crosstalk between distinct histone marks *in vivo*. At this level, we were able to identify only three, probably the most abundant phospho-modified residues for both H3.1 and H3.3, namely Ser-28, Ser-32, and Thr-45 (data not shown). Subsequent experiments included phosphopeptide enrichment prior LC-MS/MS analysis. This led to a dramatic increase in the number of detected phosphorylation sites specific to *P. falciparum* histones (Table 1 and S1). Two very recent studies analysed the general phosphoproteome of *P. falciparum* [17], [18] and one of these studies reported several histone phosphorylation marks in late schizonts [17]. Only a fraction of these reported modifications overlap with the phospho marks identified in the present work (Table 1 and S1). Conversely, other modifications reported only by that study were also identified in our LC-MS/MS analysis but did not pass our rigorous filter (see Experimental Procedures). It remains unclear if the differences observed in both studies are due to the fact that late schizont parasites show a distinct histone phospho-marks compared to younger parasite stages (rings and trophozoites in this study) or is due to different protein extraction methods.

Histone modifications can be recognized by nonhistone proteins with domains specific for methylated lysines, acetylated lysines or phosphorylated serines. These histone readers can recruit other proteins such as histone-modifying enzymes and promote different biological outcomes for the DNA linked to this chromatin region. Different histone phosphorylation binding modules such as 14-3-3 and BRCT are known. The 14-3-3 proteins have been reported to bind histone H3 phosphorylated at Ser-10 and/or Ser-28 residues. They have been implicated in diverse roles of regulating chromatin remodeling, transcription activation and hence gene regulation, though the exact mechanism behind these function remains elusive (reviewed in [16]). Based on our finding of frequent histone phosphorylation in *P. falciparum*, we searched for plasmodial proteins containing 14-3-3 domains, which have been reported to bind histone H3 phosphorylated at Ser-10 and/or Ser-28 residues. Two putative 14-3-3 proteins are highly expressed during the asexual parasite cycle [47], [48]. Sequence alignment of these proteins to 14-3-3 proteins from other model organisms indicated conservation of residues important for the interaction of these proteins with phosphorylated proteins or peptides. Residues responsible for dimer formation are also completely conserved for Pf14-3-3I, but only partially conserved for Pf14-3-3II (Figure 3) [44], [49].

We also detected H3S10ph and H3S28ph modifications in our purified samples. Hence, we produced GST-tagged recombinant versions of these two putative parasite 14-3-3 proteins, which we named Pf14-3-3I and Pf14-3-3II, to analyse their histone binding properties. In an ELISA-based binding assay, both of these proteins bound purified parasite histones, with 14-3-3I binding distinctively to the H3S28ph peptide, and not the H3S10ph peptide, demonstrating selectivity for one phospho-mark over the other. The canonical 14-3-3 binding motif includes a proline residue at position P+2 from the phosphoserine [35], [37]. This proline introduces a turn in the phosphopeptide that allows the remaining protein to exit the 14-3-3 binding pocket [50]. Both *P. falciparum* histone H3.1 and H3.3 have a proline at P+2 after S28, the canonical 14-3-3 binding motif. Conversely, *P. falciparum* histone H3.1 and H3.3 contain motifs ARKSTAG and ARKSTGG, respectively, in the vicinity of S10. The GG sequence in H3.3 could allow such a turn but the AG sequence in H3.1 might not allow a similar degree of flexibility. The differences in these potential 14-3-3 binding motifs might explain the differential Pf14-3-3 histone/peptide binding results. In yeast, 14-3-3 proteins preferentially recognize H3S10phK14ac over H3S10ph [39], demonstrating how neighbouring modifications can affect binding of these proteins. Hence, we tested neighbourhood effect by including dually modified peptides H3S10phK14ac and H3S28phS32ph in the study. Recombinant 14-3-3I clearly bound H3S28phS32ph peptide but did not bind to H3S10phK14ac peptide in our *in vitro* studies. This demonstrated that neighbouring modifications did not affect the binding pattern of the protein. However, even though Pf14-3-3II bound purified parasite histones, it did not preferentially bind to any of the H3 peptides included in this study, while demonstrating low level of binding to all the peptides. This leads us to speculate that Pf14-3-3II might be recognizing some specific combination of modifications retained in purified parasite histones that are not represented in our synthetic peptides. Alternatively, as the residues responsible for dimer formation are only partially conserved for Pf14-3-3II it remains possible that this protein functions as a heterodimer with either Pf14-3-3I or other presently unidentified proteins. The 14-3-3 proteins are known to function as both homo- and heterodimers [49]. Further experiments need to be done to confirm whether Pf14-3-3II is another member of the histone mark reading machinery and to what extent, if any, protein dimerization plays a role in that function. Additionally, these Pf14-3-3 proteins may be subject to a structure-based auto-inhibitory mechanism. Structural modelling using the I-TASSER server resulted in predicted Pf14-3-3 structures that contain C-terminal protein segments located in the canonical 14-3-3 phosphopeptide binding site (Figure S2), as has been shown for the recently solved *C. parvum* 14-3-3 protein [51]. This C-terminal region has been implicated in interfering with 14-3-3 ligand binding through folding back into the peptide binding pocket, providing a regulatory mechanism of 14-3-3 effector function [52]. Strikingly, all five predicted structural models of Pf14-3-3II included a portion of their C-terminus in the phosphoprotein binding-pocket.

14-3-3 proteins are involved in the regulation of subcellular localization, activation or inhibition of enzymes, and signal transduction [53]. Consistent with this pleiotropic role, immunolocalization analysis located Pf114-3-3I in cytoplasmic and nuclear compartments. Additionally, rodent malaria 14-3-3 proteins have been shown to interact, in a phospho-dependent manner, with the internalized host skeletal protein dematin and it might determine the localization of host-derived dematin inside the parasite [44]. To further explore the biological role of Pf14-3-3 proteins, co-immunoprecipitation experiments may identify their interaction partners and chromatin immunoprecipitation assays may determine the chromatin occupation sites of these proteins and reveal a functional link to gene transcription or cell division.

In conclusion, our data set the framework for studies on histone phosphorylation mediated regulatory processes in chromatin biology of malaria parasites. This work opens up avenues to study signal transduction cascades leading to histone phosphorylation and ultimately controlling transcription and other nuclear processes in malaria parasites.

## Supporting Information

Figure S1 **S**equence alignment between different plasmodium core histones and their variant: core histone H2A (PFF0860c), H2B (PF11_0062), and H3 (PFF0510w) and their variants H2A.Z (PFC0920w), H2B.Z (PF07_0054), and H3.3 (PFF0865w). Histone variant H2B.Z correspond to the previously named H2Bv.
(TIF)

Figure S2 Overlay of homology-based structural models of Pf14-3-3 proteins. All five Pf14-3-3I (A) and Pf14-3-3II (B) structural models returned from the I-TASSER server are shown in different colours.
(TIF)

Figure S3 Annotated Mass Spectra for H2AS18ph.
(JPG)

Figure S4 Annotated Mass Spectra for H2AS120ph.
(JPG)

Figure S5 Annotated Mass Spectra for H2AS120phT126ph.
(JPG)

Figure S6 Annotated Mass Spectra for H2AT126ph.
(JPG)

Figure S7 Annotated Mass Spectra for H2BS104ph.
(JPG)

Figure S8 Annotated Mass Spectra for H2B.ZS1ph.
(JPG)

Figure S9 Annotated Mass Spectra for H2A.ZS32ph.
(JPG)

Figure S10 Annotated Mass Spectra for H3.1S10ph.
(JPG)

Figure S11 Annotated Mass Spectra for H3.3S10ph.
(JPG)

Figure S12 Annotated Mass Spectra for H3.1S22ph.
(JPG)

Figure S13 Annotated Mass Spectra for H3.1S28ph.
(JPG)

Figure S14 Annotated Mass Spectra for H3.3S28ph.
(JPG)

Figure S15 Annotated Mass Spectra for H3.1S32ph.
(JPG)

Figure S16 Annotated Mass Spectra for H3.3S32ph.
(JPG)

Figure S17 Annotated Mass Spectra for H3.1S57ph.
(JPG)

Figure S18 Annotated Mass Spectra for H3.3S57ph.
(JPG)

Figure S19 Annotated Mass Spectra for H3.1T11ph.
(JPG)

Figure S20 Annotated Mass Spectra for H3.3T11ph.
(JPG)

Figure S21 Annotated Mass Spectra for H3T45ph_H3.1_H3.3.
(JPG)

Table S1 List of all histone phospho-modifications identified in this study on peptides with a significant score (Mascot score >20) using MaxQuant. Only the peptides that passed filter requiring site probability score >0.75 and a difference score >5 are listed in table 1.
(XLS)
