# Supplementary figures and images for: Comprehensive Histone Phosphorylation Analysis and Identification of Pf14-3-3 Protein as a Histone H3 Phosphorylation Reader in Malaria Parasites
Source: PLoS One. 2013 Jan 7;8(1):e53179. doi: 10.1371/journal.pone.0053179 (PMC3538786; doi:10.1371/journal.pone.0053179)

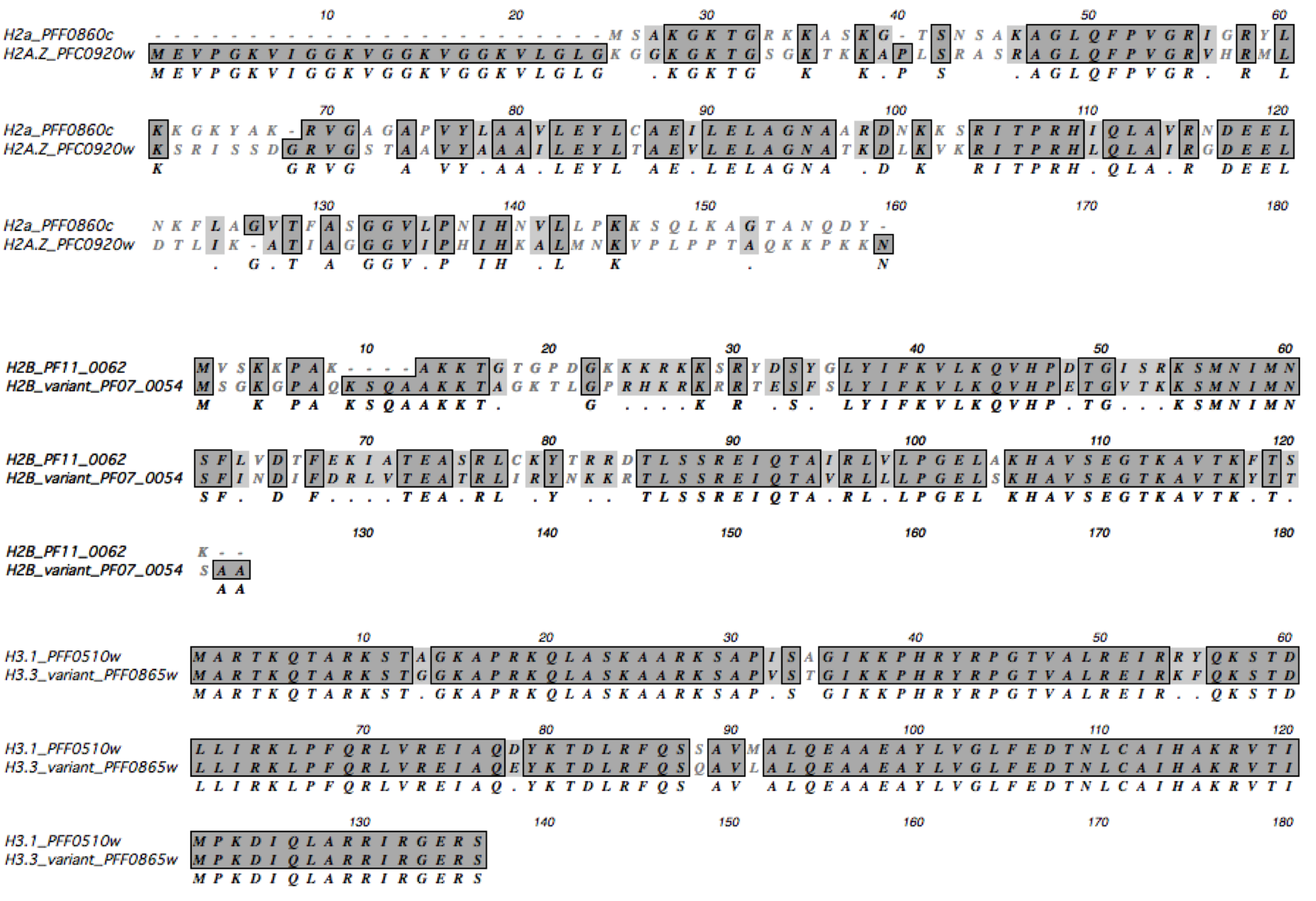

Supplement: Figure S1 — Sequence alignment between different plasmodium core histones and their variant: core histone H2A (PFF0860c), H2B (PF11_0062), and H3 (PFF0510w) and their variants H2A.Z (PFC0920w), H2B.Z (PF07_0054), and H3.3 (PFF0865w). Histone variant H2B.Z correspond to the previously named H2Bv. (TIF) [file pone.0053179.s001.tif]

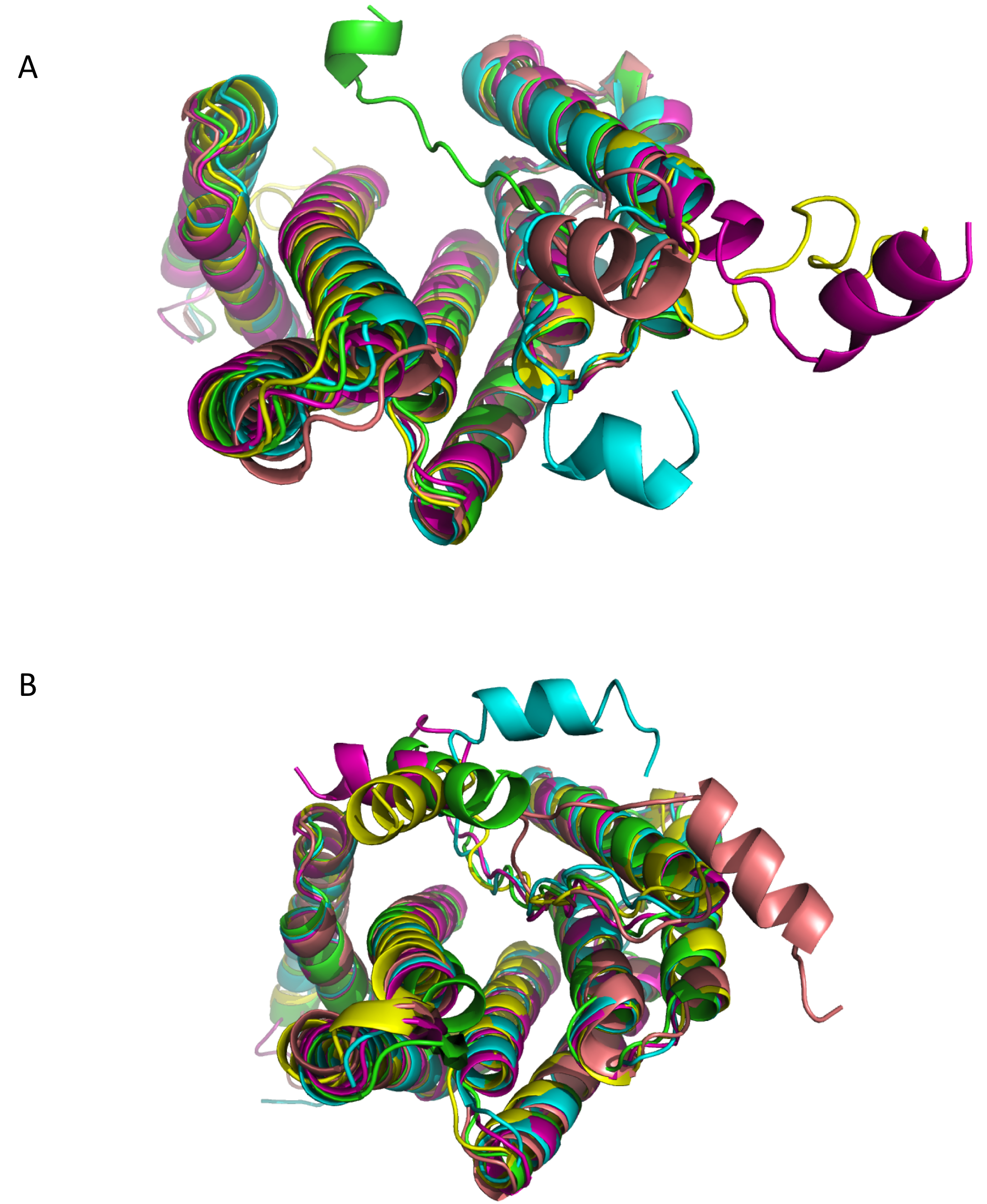

Supplement: Figure S2 — Overlay of homology-based structural models of Pf14-3-3 proteins. All five Pf14-3-3I (A) and Pf14-3-3II (B) structural models returned from the I-TASSER server are shown in different colours. (TIF) [file pone.0053179.s002.tif]

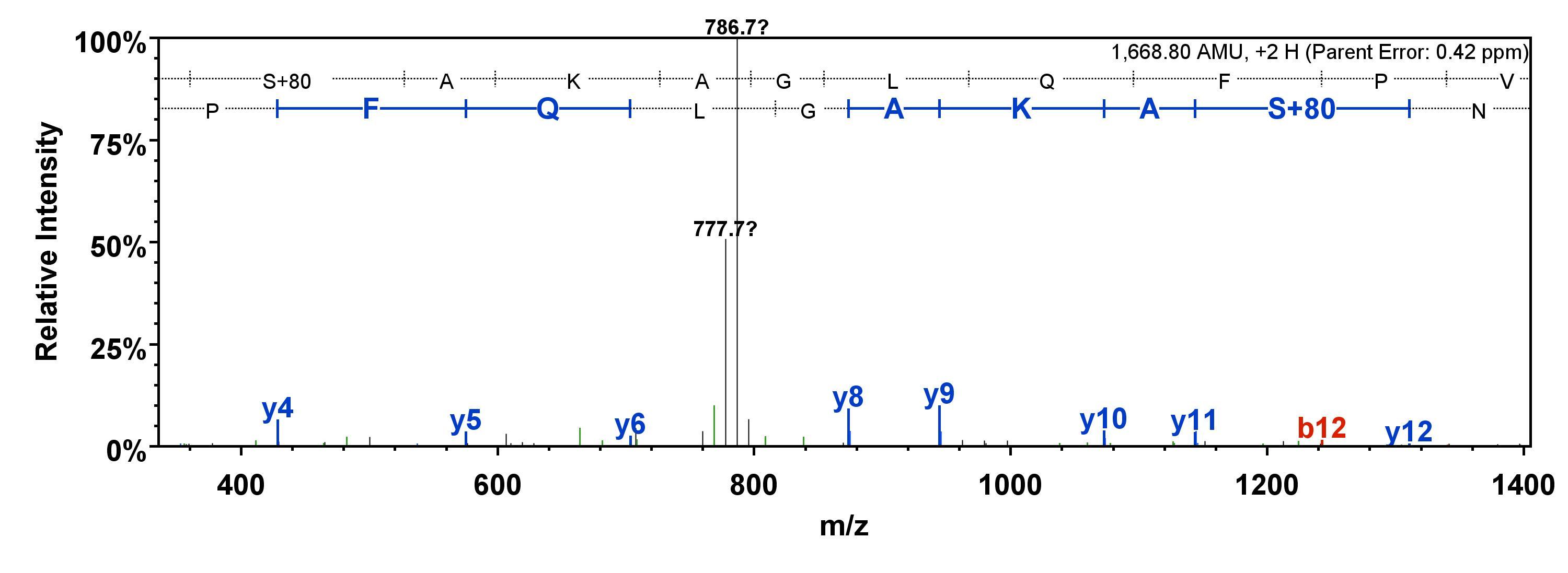

Supplement: Figure S3 — Annotated Mass Spectra for H2AS18ph. (JPG) [file pone.0053179.s003.jpg]

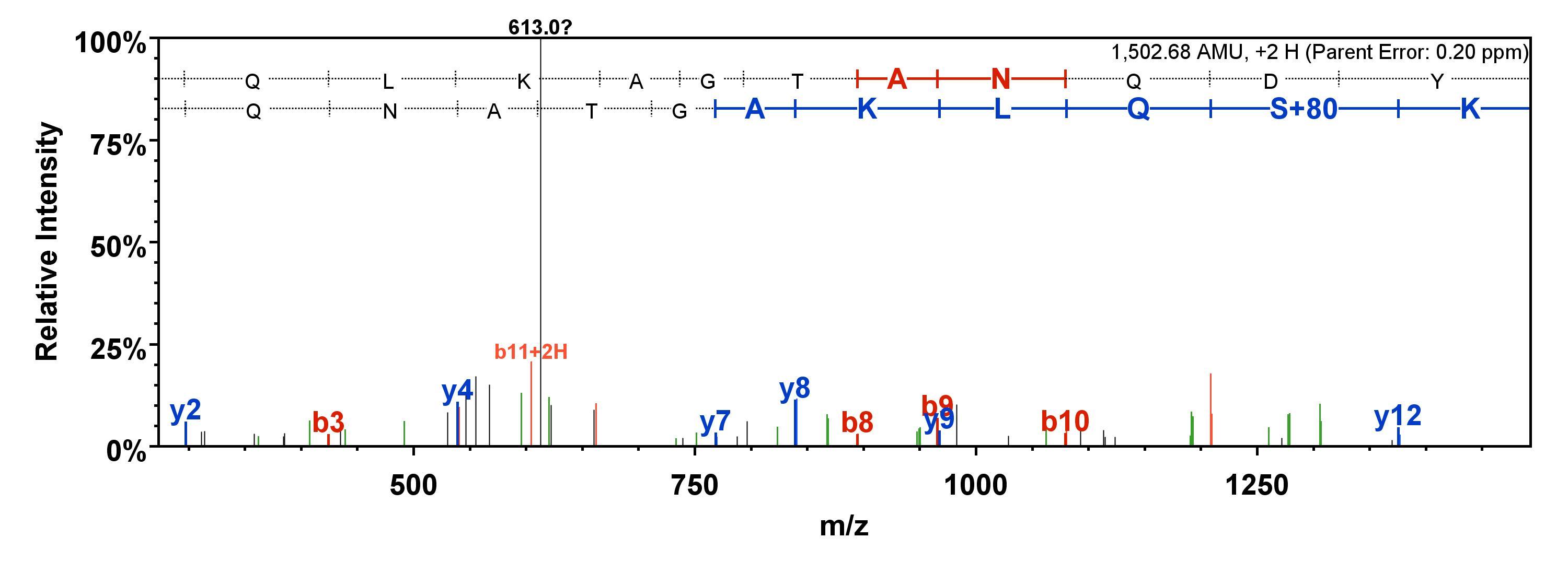

Supplement: Figure S4 — Annotated Mass Spectra for H2AS120ph. (JPG) [file pone.0053179.s004.jpg]

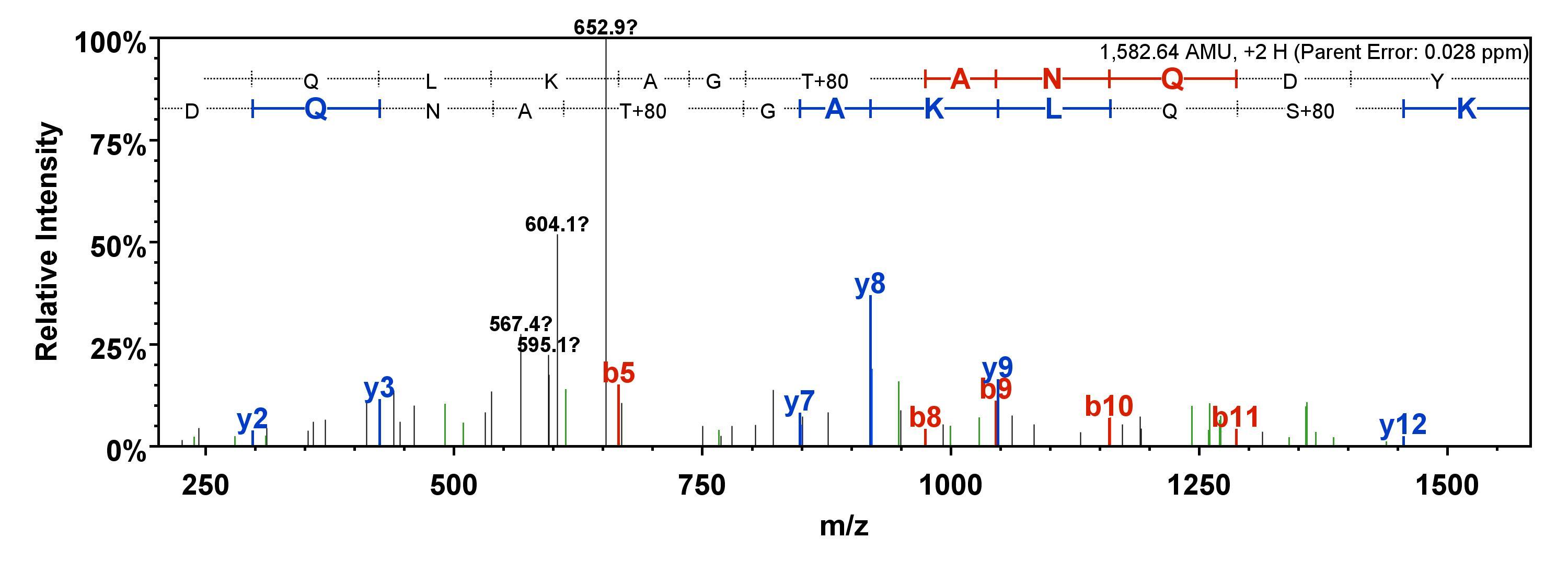

Supplement: Figure S5 — Annotated Mass Spectra for H2AS120phT126ph. (JPG) [file pone.0053179.s005.jpg]

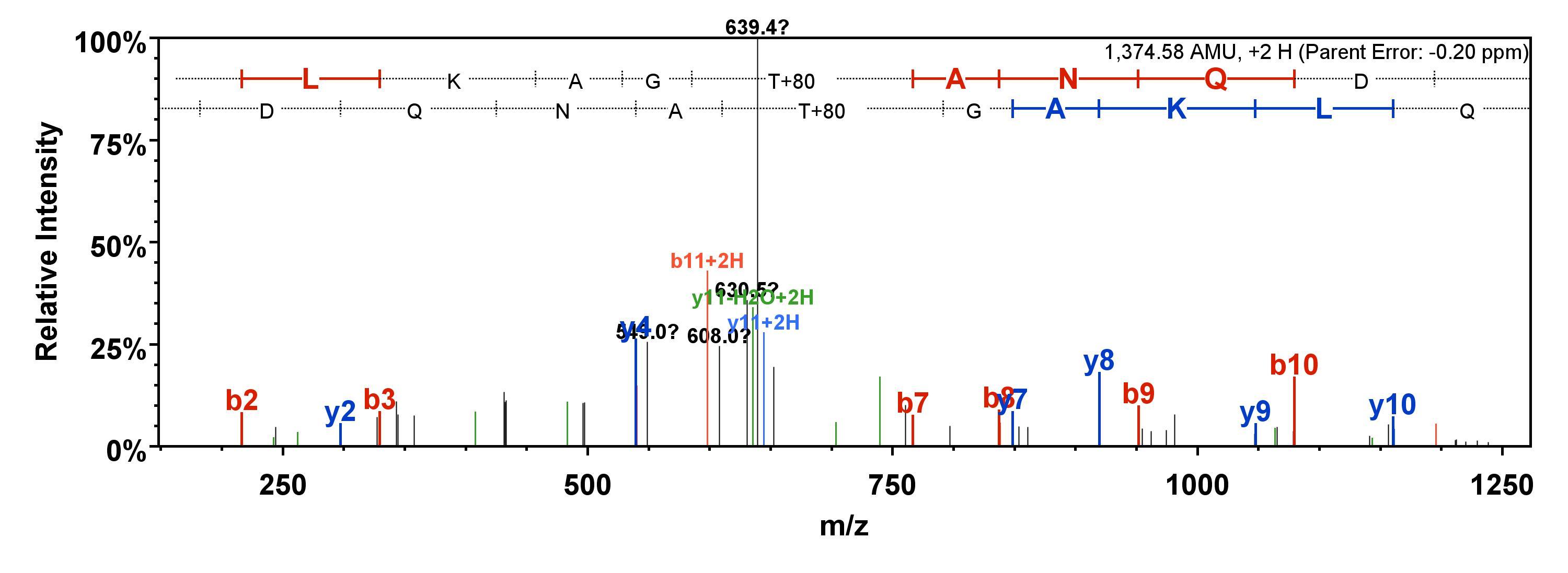

Supplement: Figure S6 — Annotated Mass Spectra for H2AT126ph. (JPG) [file pone.0053179.s006.jpg]

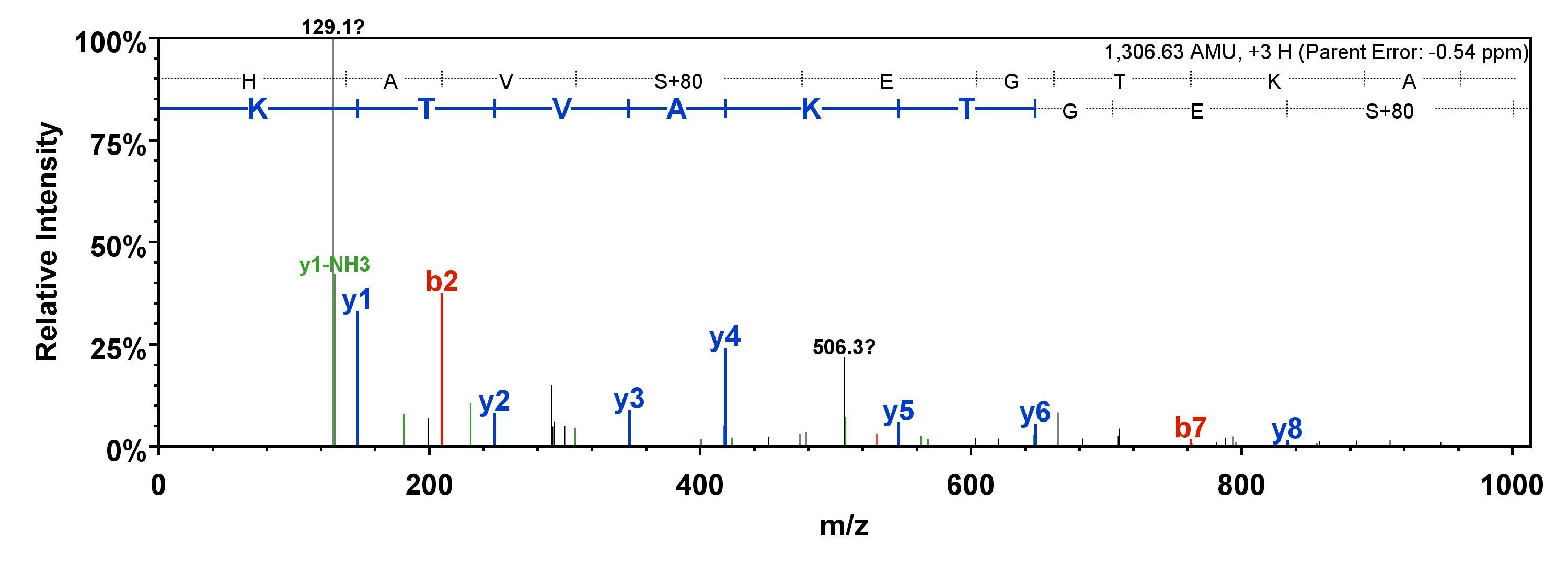

Supplement: Figure S7 — Annotated Mass Spectra for H2BS104ph. (JPG) [file pone.0053179.s007.jpg]

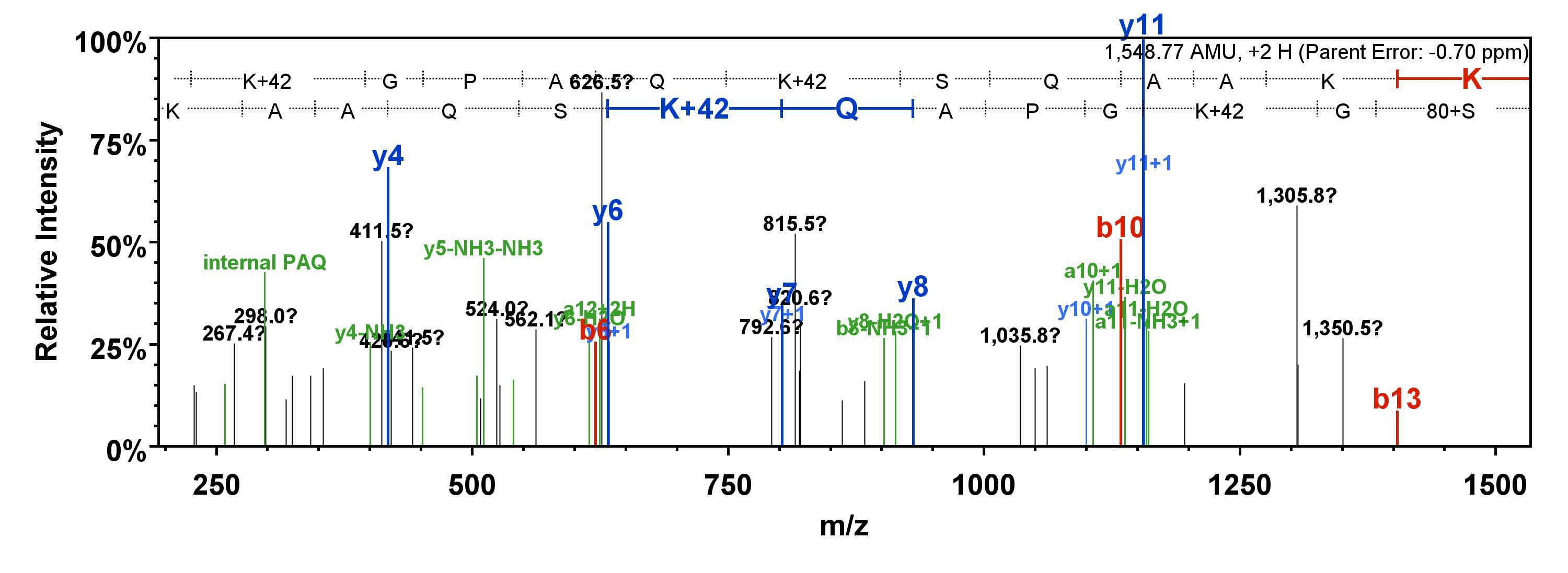

Supplement: Figure S8 — Annotated Mass Spectra for H2B.ZS1ph. (JPG) [file pone.0053179.s008.jpg]

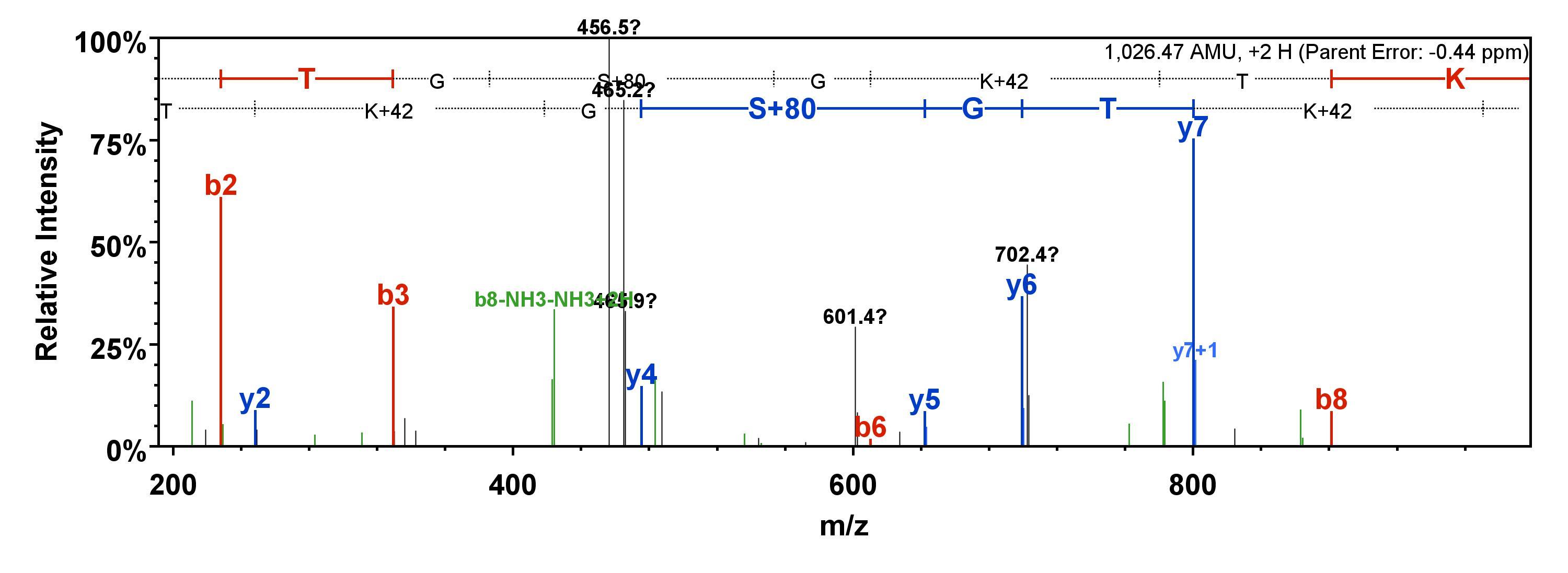

Supplement: Figure S9 — Annotated Mass Spectra for H2A.ZS32ph. (JPG) [file pone.0053179.s009.jpg]

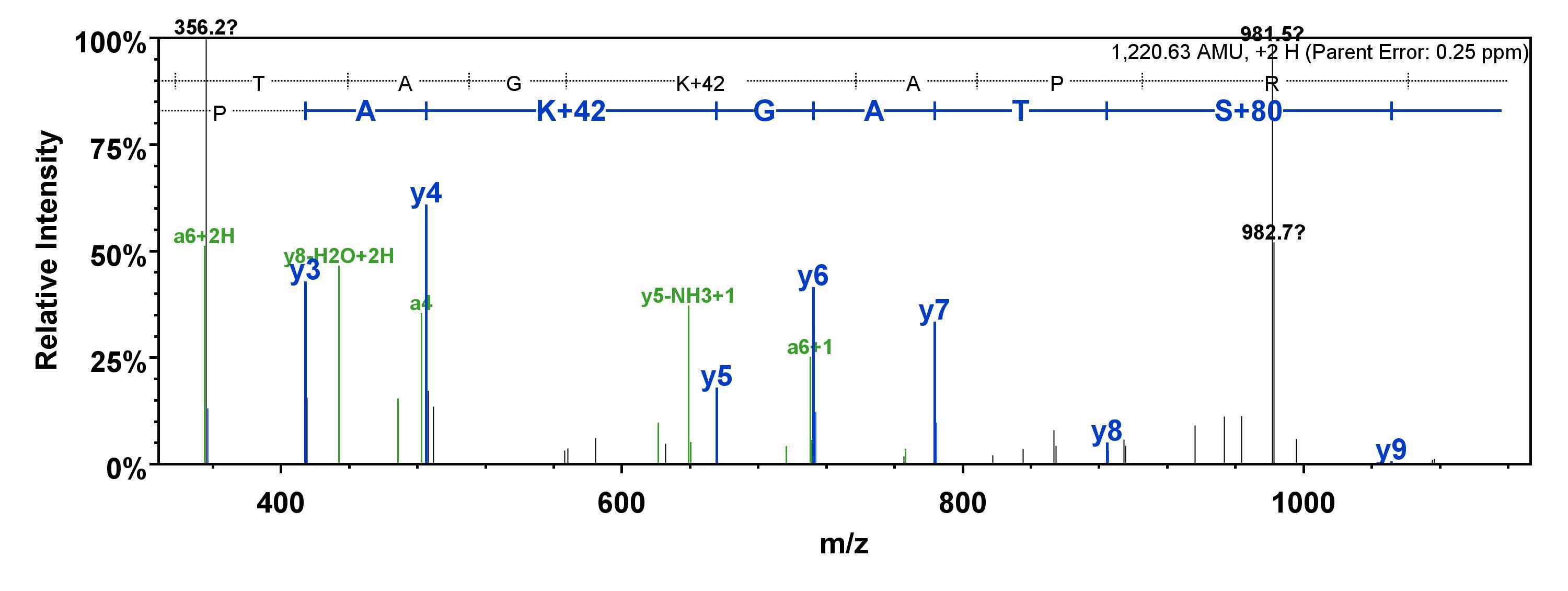

Supplement: Figure S10 — Annotated Mass Spectra for H3.1S10ph. (JPG) [file pone.0053179.s010.jpg]

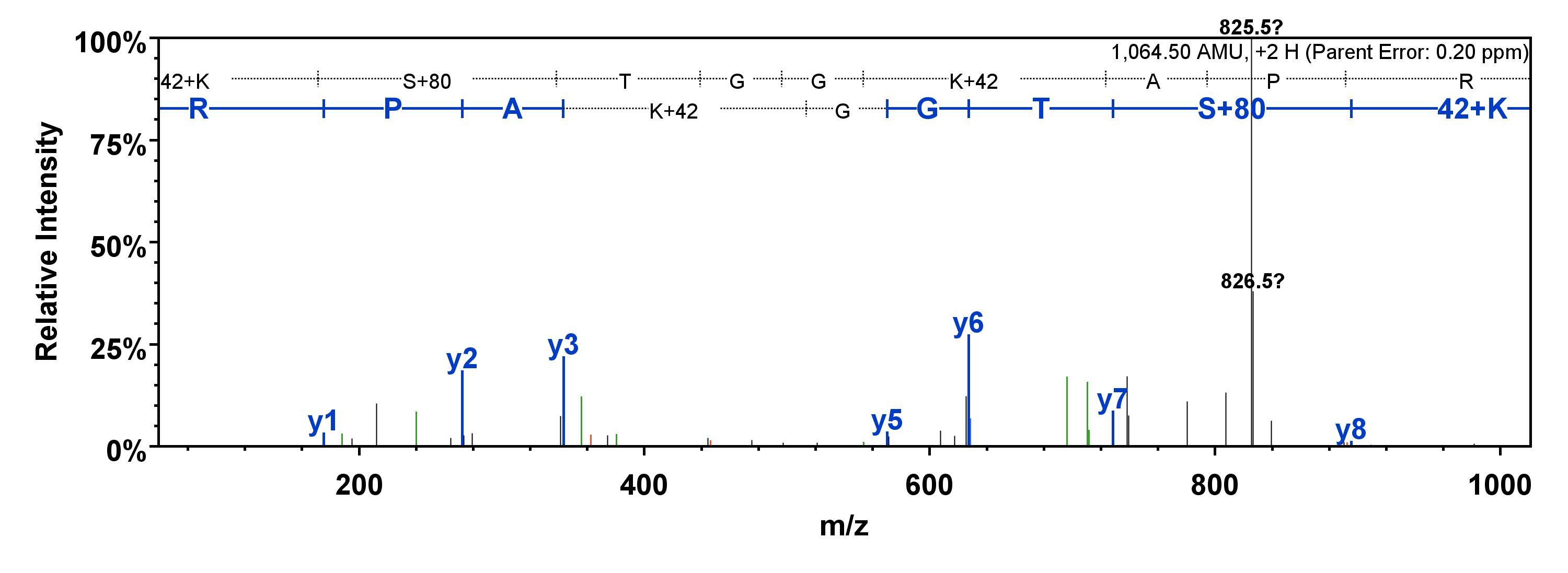

Supplement: Figure S11 — Annotated Mass Spectra for H3.3S10ph. (JPG) [file pone.0053179.s011.jpg]

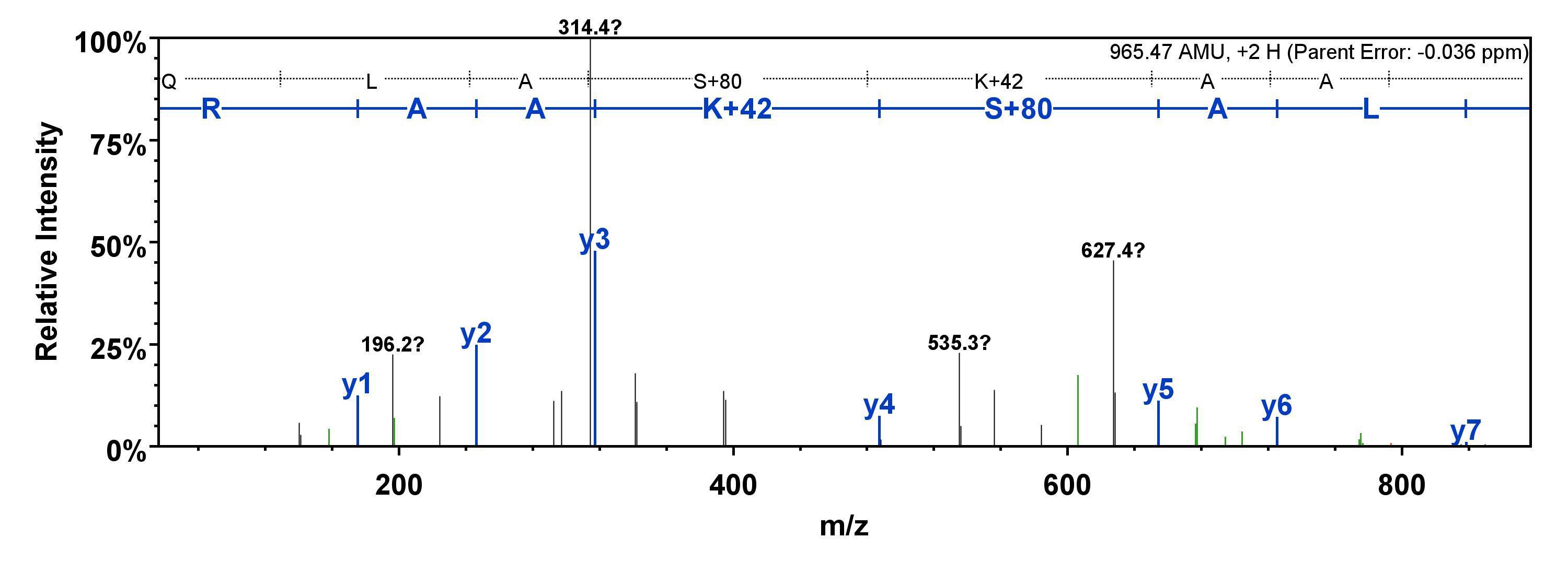

Supplement: Figure S12 — Annotated Mass Spectra for H3.1S22ph. (JPG) [file pone.0053179.s012.jpg]

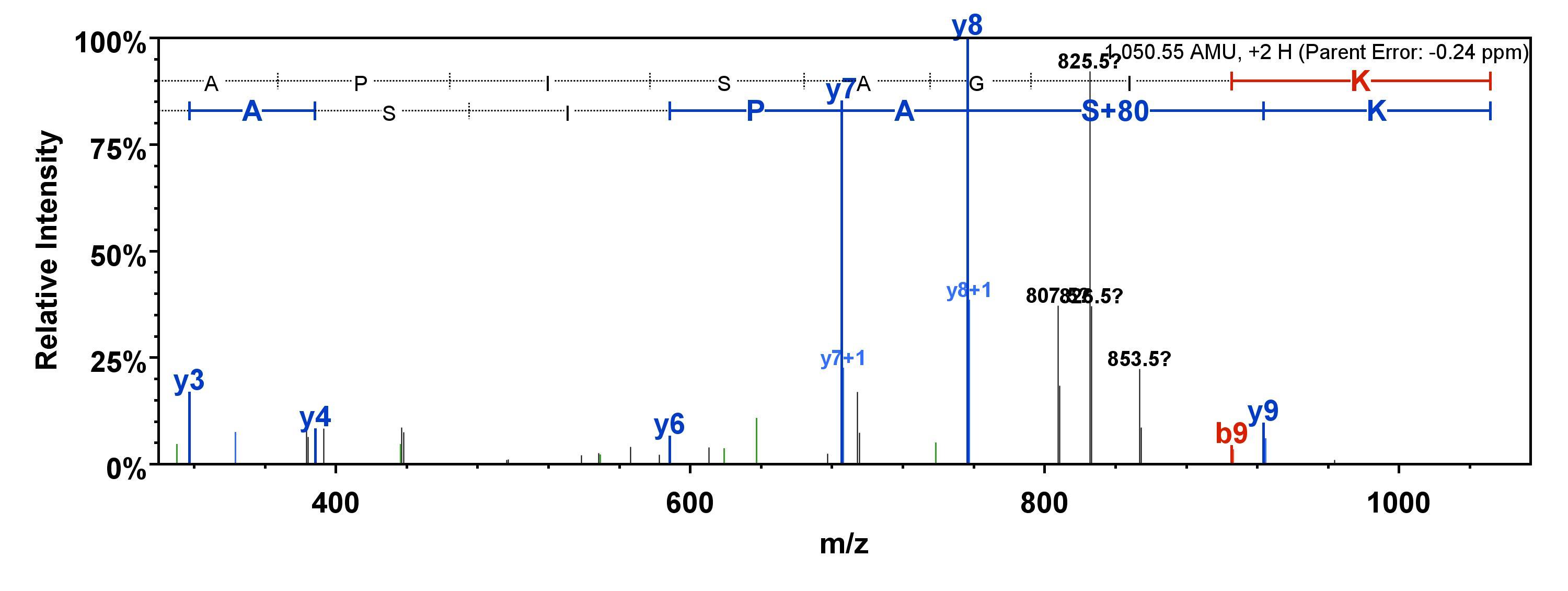

Supplement: Figure S13 — Annotated Mass Spectra for H3.1S28ph. (JPG) [file pone.0053179.s013.jpg]

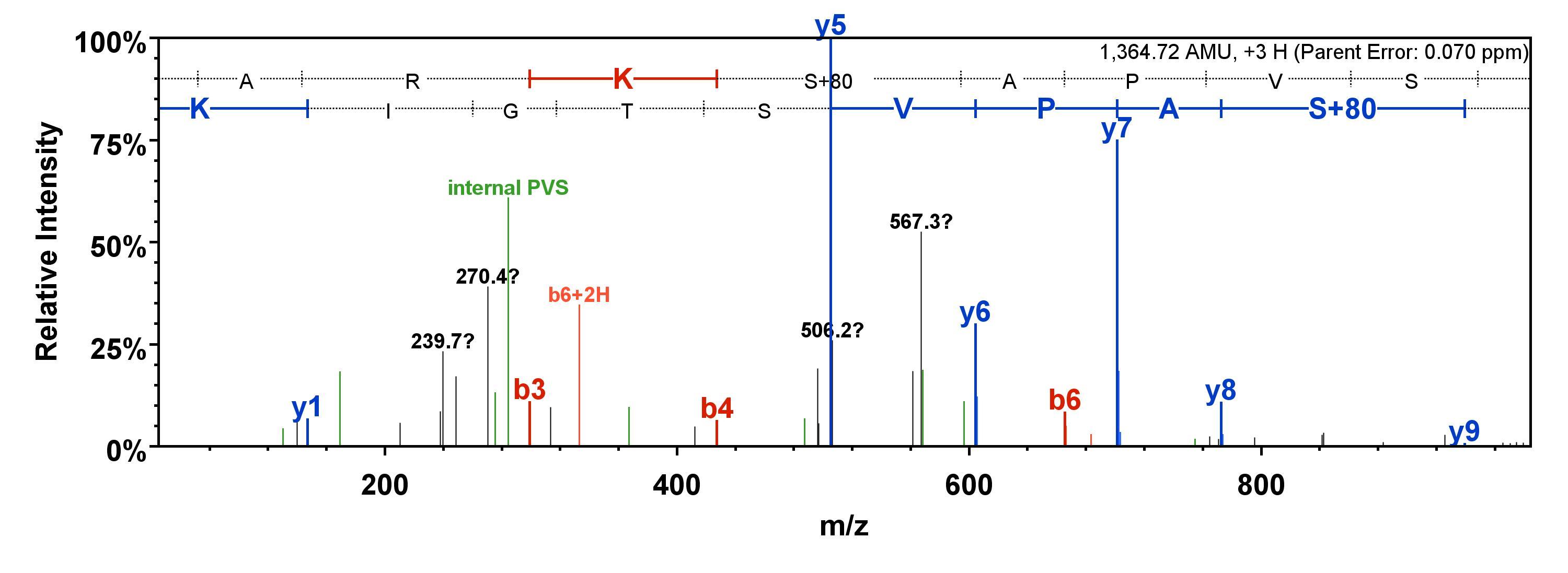

Supplement: Figure S14 — Annotated Mass Spectra for H3.3S28ph. (JPG) [file pone.0053179.s014.jpg]

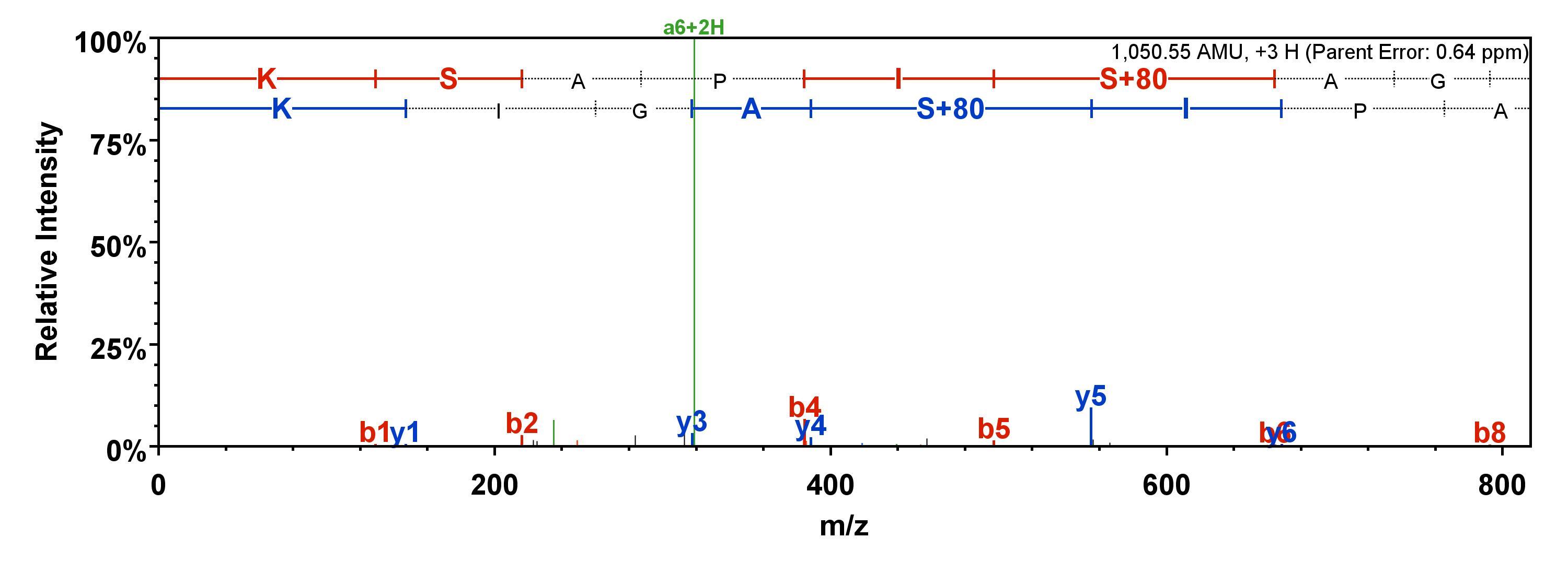

Supplement: Figure S15 — Annotated Mass Spectra for H3.1S32ph. (JPG) [file pone.0053179.s015.jpg]

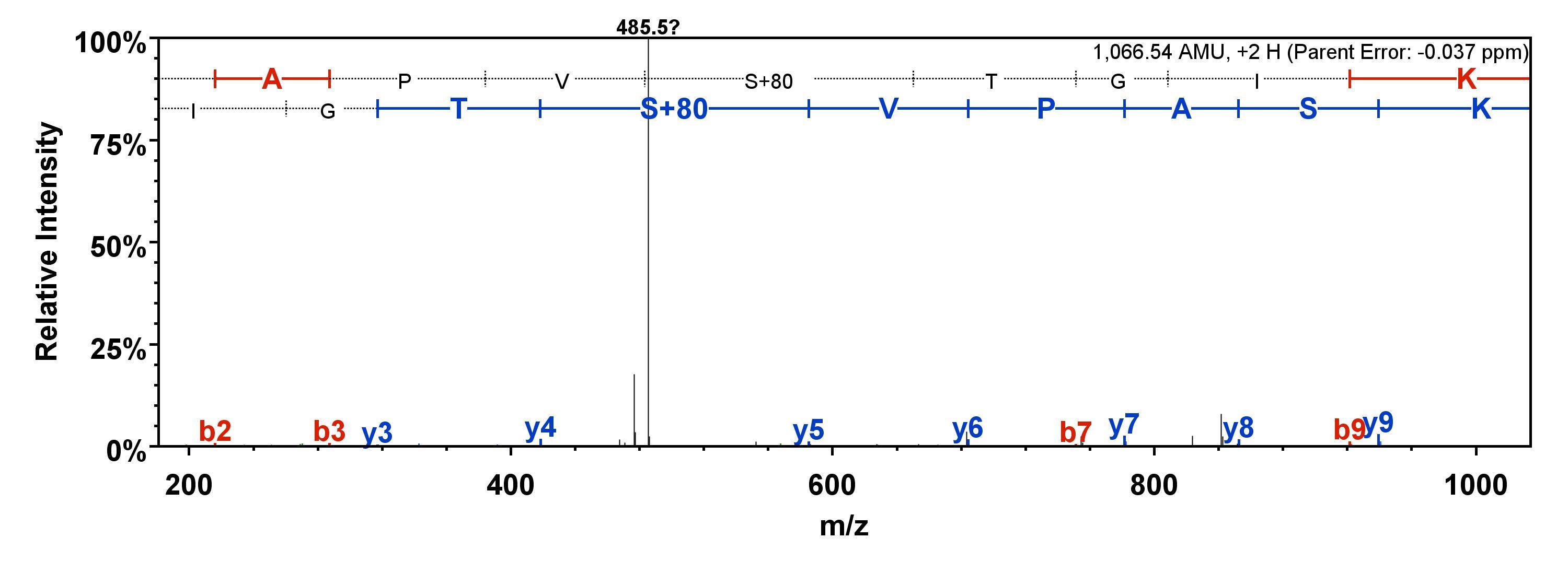

Supplement: Figure S16 — Annotated Mass Spectra for H3.3S32ph. (JPG) [file pone.0053179.s016.jpg]

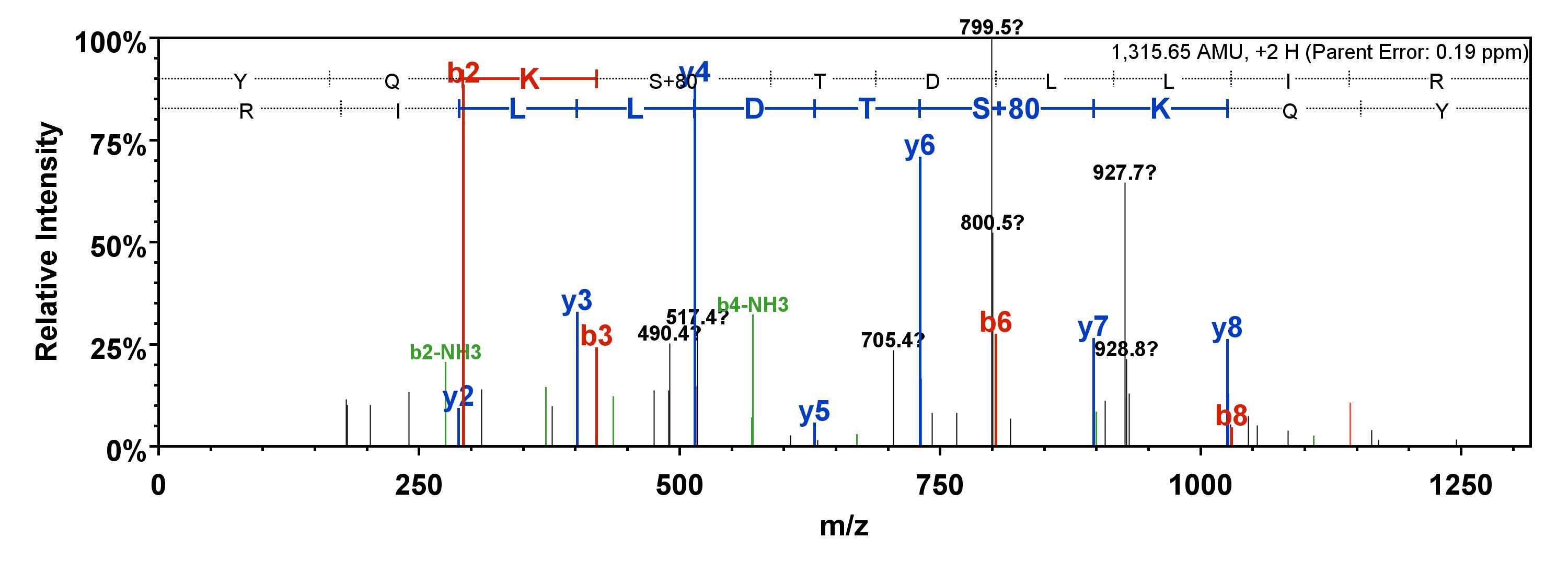

Supplement: Figure S17 — Annotated Mass Spectra for H3.1S57ph. (JPG) [file pone.0053179.s017.jpg]

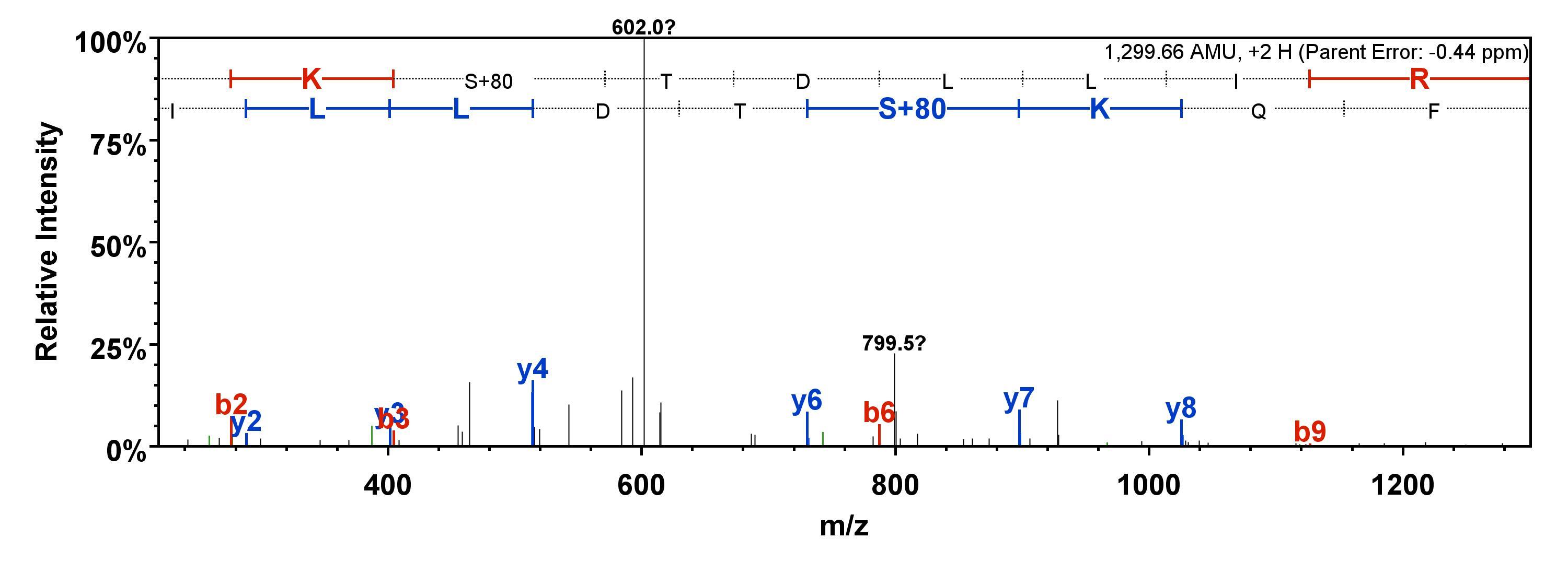

Supplement: Figure S18 — Annotated Mass Spectra for H3.3S57ph. (JPG) [file pone.0053179.s018.jpg]

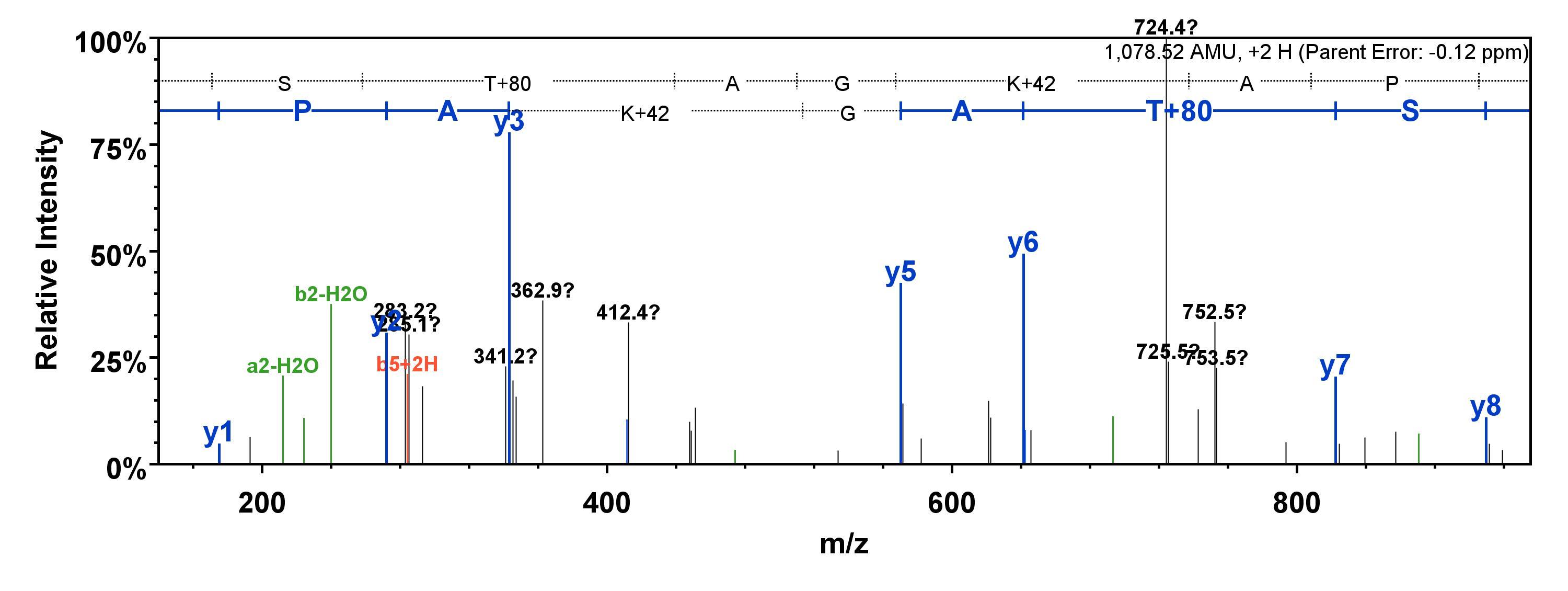

Supplement: Figure S19 — Annotated Mass Spectra for H3.1T11ph. (JPG) [file pone.0053179.s019.jpg]

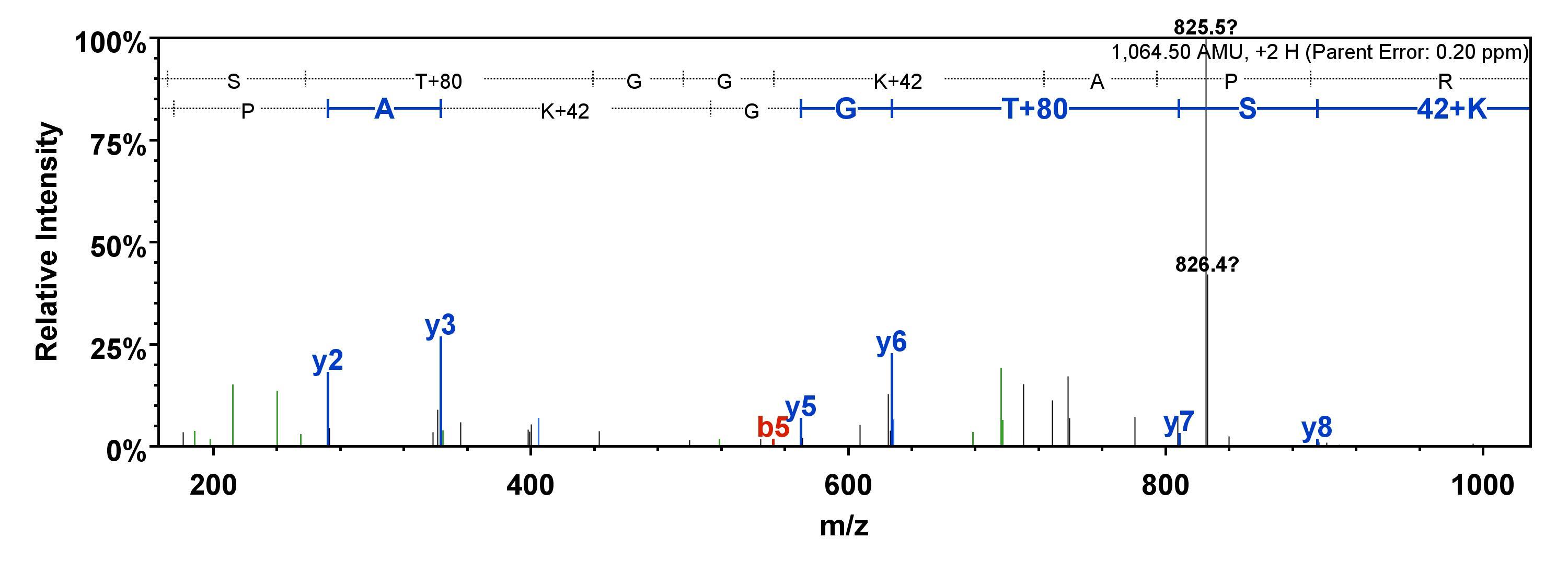

Supplement: Figure S20 — Annotated Mass Spectra for H3.3T11ph. (JPG) [file pone.0053179.s020.jpg]

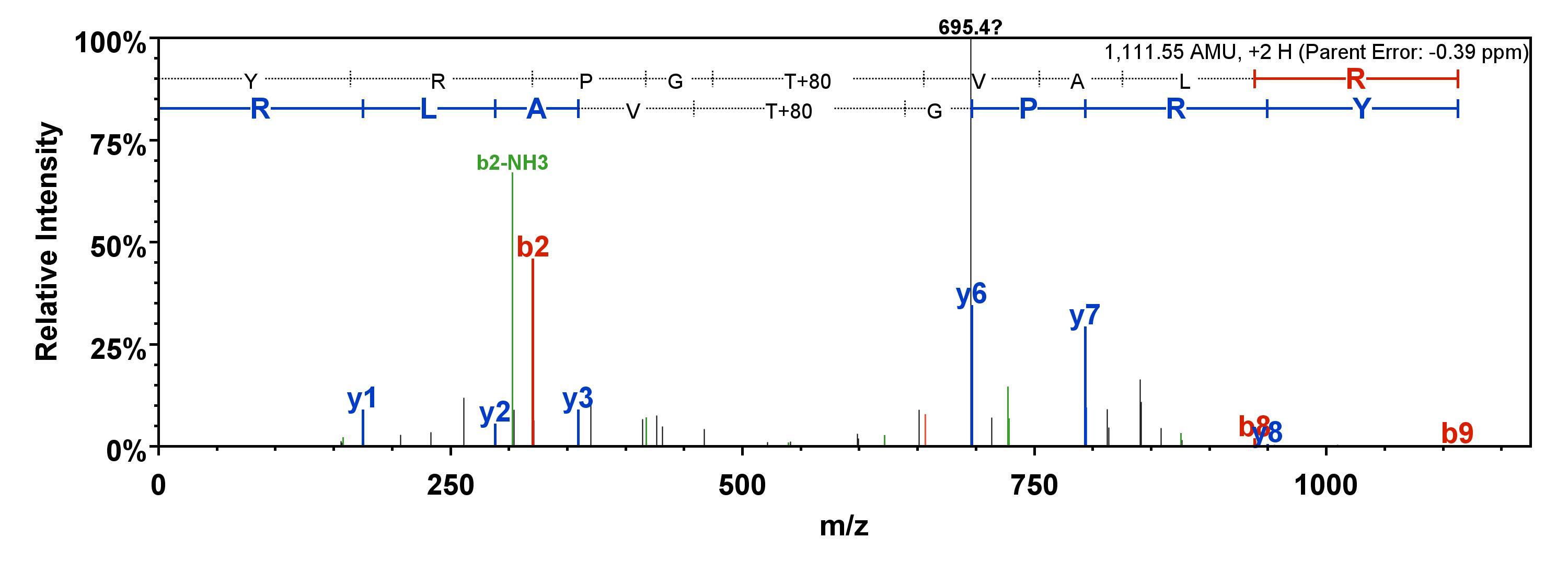

Supplement: Figure S21 — Annotated Mass Spectra for H3T45ph_H3.1_H3.3. (JPG) [file pone.0053179.s021.jpg]
